# Retromer promotes the lysosomal turnover of mtDNA

**DOI:** 10.1126/sciadv.adr6415

**Published:** 2025-04-04

**Authors:** Parisa Kakanj, Mari Bonse, Arya Kshirsagar, Aylin Gökmen, Felix Gaedke, Ayesha Sen, Belén Mollá, Elisabeth Vogelsang, Astrid Schauss, Andreas Wodarz, David Pla-Martín

**Affiliations:** ^1^ Institute of Genetics, University of Cologne, Cologne, Germany.; ^2^ Cologne Excellence Cluster on Cellular Stress Response in Aging-Associated Diseases (CECAD), University of Cologne, Cologne, Germany.; ^3^ Institute of Physiology, University Clinics and Faculty of Medicine, University of Cologne, Cologne, Germany.; ^4^ Center for Molecular Medicine Cologne (CMMC), University of Cologne, Cologne, Germany.; ^5^ Institute of Biochemistry and Molecular Biology, University Clinics and Faculty of Medicine, Heinrich-Heine University Düsseldorf, Düsseldorf, Germany.; ^6^ Independent Researcher, Valencia, Spain.; ^7^ Department of Molecular Cell Biology, Institute I for Anatomy. University Clinics and Faculty of Medicine, University of Cologne, Cologne, Germany.

## Abstract

Mitochondrial DNA (mtDNA) is exposed to multiple insults produced by normal cellular function. Upon mtDNA replication stress, the mitochondrial genome transfers to endosomes for degradation. Using proximity biotinylation, we found that mtDNA stress leads to the rewiring of the mitochondrial proximity proteome, increasing mitochondria’s association with lysosomal and vesicle-related proteins. Among these, the retromer complex, particularly VPS35, plays a pivotal role by extracting mitochondrial components. The retromer promotes the formation of mitochondrial-derived vesicles shuttled to lysosomes. The mtDNA, however, directly shuttles to a recycling organelle in a BAX-dependent manner. Moreover, using a *Drosophila* model carrying a long deletion on the mtDNA (ΔmtDNA), we found that ΔmtDNA activates a specific transcriptome profile to counteract mitochondrial damage. Here, *Vps35* expression restores mtDNA homoplasmy and alleviates associated defects. Hence, we demonstrate the existence of a previously unknown quality control mechanism for the mitochondrial matrix and the essential role of lysosomes in mtDNA turnover to relieve mtDNA damage.

## Teaser

Lysosomal uptake of mtDNA controls mitochondrial quality.

## INTRODUCTION

Mitochondria are multifaceted organelles in charge of many essential cellular functions. Unique among cellular organelles, mitochondria contain their own genome, also known as mitochondrial DNA (mtDNA). Present in multiple copies per cell, the mitochondrial genome is organized within structures known as mitochondrial nucleoids. mtDNA is relatively small but encodes 13 critical proteins essential for the mitochondrial respiratory chain and a subset of RNAs and tRNAs required for mitochondrial protein synthesis. All these components are indispensable for maintaining mitochondrial function and cellular homeostasis (*1*).

One of the main subproducts generated during the coupling of the different mitochondrial pathways to produce adenosine triphosphate (ATP) are reactive oxygen species (ROS) (*2*). ROS, including superoxide anions, hydrogen peroxide, and hydroxyl radicals, are generated when electrons leak from the electron transport chain and react with oxygen molecules. ROS damages not only proteins and lipids but also the mtDNA, modifying the biochemical properties of the molecule, altering base-pairing properties, and affecting the maintenance of the mitochondrial genome, serving as a source for mutations (*3*, *4*). These mutations impair respiratory chain complexes, increasing oxidative stress and triggering a self-amplifying cycle that results in mitochondrial dysfunction. Thus, the progressive accumulation of mtDNA damage influences many human diseases, accelerates aging, and, in marked cases, even leads to cellular death (*5*).

From energy production and ion exchange to metabolite synthesis, all mitochondrial functions depend on maintaining the integrity of the network through mitochondrial turnover. Since the discovery of selective degradation of mitochondria in mammals in the early 21st century, numerous parallel pathways have been identified (*6*). The PINK1-Parkin autophagy pathway targets large mitochondrial segments for degradation in response to acute damage or in situations requiring mitochondrial adaptation, such as during cell differentiation or changes in nutrient availability (*7*). Other complementary pathways work in parallel to selectively remove only dysfunctional mitochondrial pieces, thereby preserving the healthy portions of the network and maintaining cellular homeostasis (*6*).

The presence of mtDNA in autophagy-related structures, independent of other mitochondrial parts, suggests a specific selective quality control pathway for the mitochondrial genome (*8*). Several mechanisms have been proposed to explain how mtDNA is degraded in response to stress, including mitochondrial-derived vesicles (MDVs) (*9*) and piecemeal mitophagy (*10*, *11*). Nevertheless, the exact mechanism seems to depend on the initial stress. Thus, upon fumarate accumulation, the mitochondrial genome appears encapsulated in TOM20−/PDH+ MDVs (*9*). Upon antimycin/oligomycin stress, the inner membrane and, eventually, the mtDNA are eliminated through lysosomal-dependent piecemeal mitophagy through VDAC1 pores (*11*). Furthermore, cells treated with low levels of ethidium bromide separate mitochondrial nucleoids into inner membrane subdomains, which, upon fission, are eliminated in autophagosome-dependent piecemeal mitophagy (*10*). In this context, the mitochondrial inner membrane PHB2 serves as a mitophagy receptor after the degradation of the outer mitochondrial membrane (*10*, *12*). Independent of the specificities of each path, all of them point toward the separation of a mitochondrial piece and its degradation in recycling organelles.

Interfering with mtDNA replication leads to the accumulation of oxidative damage in the mitochondrial genome (*13*). The biochemical changes initiated by ROS modify the architecture of the mitochondrial membranes and allow the translocation of the mtDNA to the endosomal compartment, where it will be degraded within recycling organelles (*13*). We and others recently found that the extraction of the mtDNA responds to the coordination between mitochondrial membrane and vesicle trafficking proteins. The endosomal protein RAB5C physically interacts with Mitofusin 1 and Mitofusin 2 and assists in endosomal approach and mtDNA transfer (*14*). Similarly, proper disposal of mtDNA requires the mitochondrial membrane protein SAMM50 (*13*). Disturbance of this selective quality control pathway leads to the activation of the innate immune response with potential implications in aging and several human diseases (*14*–*16*).

In the current study, we sought to elucidate the mechanisms underlying the elimination of the mitochondrial genome upon mtDNA replication stress. By spatial proteomics of the mitochondria-endosome contact sites, we identified a group of lysosomal proteins involved in mtDNA turnover. Mitochondrial matrix components, including the mtDNA, exit the mitochondrial network assisted by the retromer, although they do not follow the same path. The accumulation of the mtDNA outside the mitochondrial compartment is BAX dependent and enhanced by lysosomal inhibition with chloroquine. Using correlative light and electron microscopy (CLEM), we demonstrate that the piecemeal removal of mtDNA from mitochondria occurs with direct transfer to a recycling organelle. The retromer assists this process by stimulating the lysosomal function and mitochondrial fragmentation. Furthermore, using a *Drosophila* model with a long deletion in the mtDNA (ΔmtDNA), we validated the role of the retromer in promoting mtDNA quality control in vivo. Our results reveal a mechanism for the degradation of the mitochondrial matrix, including the mtDNA, and highlight the lysosomes as the central organelles maintaining mitochondrial function.

## RESULTS

### mtDNA damage changes the proteome of mitochondria-endosome contact sites

**Fig. 1.**
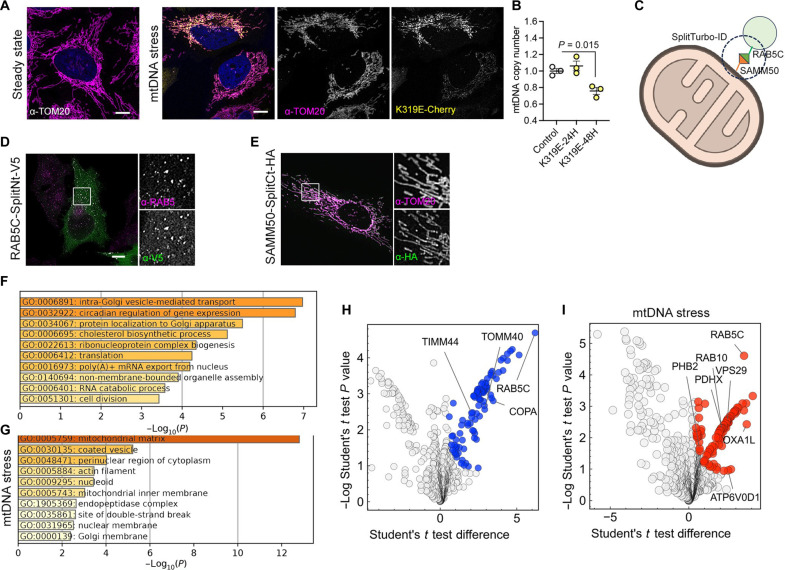
mtDNA damage follows a lysosomal-dependent degradation pathway. (**A**) Confocal images of HeLa cells in the steady state and expressing TWNK^K319E^-mCherry (mtDNA stress) labeled with α-TOM20. (**B**) mtDNA copy number analysis in HeLa cells transiently transfected with TWNK^K319E^-mCherry. (**C**) Schematic representation of the SplitTurboID assay. The early endosomal marker RAB5C and the mitochondrial outer membrane protein SAMM50 were used to determine the proximity proteome of mitochondria and endosomes. Illustration from NIAID NIH BIOART Source. (**D** and **E**) Immunostaining of HeLa cells expressing (D) RAB5C-SplitNt-V5 and (E) SAMM50-SplitCt-HA labeled with α-RAB5 and α-V5 and α-TOM20 and α-HA, respectively. (**F** and **G**) Pathway enrichment analysis with Metascape showing GO terms for proteins differentially enriched (F) in the steady state and (G) after expression of TWNK^K319E^-mCherry. (**H** and **I**) Volcano plot showing proteins enriched after biotinylation and purification of cells expressing (H) SplitTurboID plasmids and (I) TWNK^K319E^-mCherry. Differentially expressed proteins compared with cells transduced only with SAMM50-SplitCt-HA (significant: *q* value < −0.05 and absolute log_2_ FC > 1) are highlighted in blue (steady state) or red (mtDNA stress) (*n* = 3 samples per group). *P* values were calculated using one-way ANOVA with Tukey correction for multiple comparisons. Scale bars, 10 μm. Data are presented as means ± SEM.

Disrupting the mtDNA replication machinery leads to the transfer of mitochondrial nucleoids to the endosomal compartment (*13*). To specifically impair mtDNA replication, we expressed a dominant negative mutation of the mitochondrial helicase Twinkle (TWNK^K319E^) in HeLa cells (Fig. 1A). The equivalent mutation in mice (TWNK^K320E^) has been validated as a reliable tool to induce mtDNA alterations, oxidative stress, and replication defects (*17*–*19*). Expression of TWNK^K319E^ reduced mtDNA copy number 48 hours posttransfection (Fig. 1B).

To detect regulators involved in this pathway, we used the promiscuous biotinylation enzyme TurboID (*20*). For specific detection of the mitochondria-endosome proteome, we used Split-TurboID (Fig. 1C). Thus, we fused the N-terminal part of TurboID to RAB5C (TurboID amino acids 1 to 72; RAB5C-SplitTurboNt-V5) and the C-terminal part to SAMM50 (TurboID amino acids 73 to 246; SAMM50-SplitTurboCt-HA), two proteins related to the endosomal disposal of the mtDNA (*13*, *14*). The expected subcellular localization of both transgene-encoded fusion proteins at endosomes and mitochondria, respectively, was validated in HeLa cells with confocal microscopy (Fig. 1, D and E).

To isolate a high number of biotinylated proteins, we generated human embryonic kidney (HEK) 293 stable cell lines expressing Split-TurboID plasmids by lentiviral transduction. Reconstitution of biotinylation activity was confirmed by Western blot and immunofluorescence (fig. S1, A and B). We noticed that cells expressing only SAMM50-SplitTurboCt-HA displayed residual enzymatic activity (fig. S1A). Therefore, to minimize potential background interference, we used these cells as a negative control and cells expressing both SAMM50-SplitTurboCt-HA and RAB5C-SplitTurboNt-V5 as the experimental approach.

The proximity proteome of the mitochondria and endosomes in the steady state showed enrichment of proteins involved in intra-Golgi vesicle transport and mitochondrial protein import (Fig. 1, F and H, and data S1). On the contrary, mtDNA replication stress elicited a complete rewiring of the mitochondria-endosome proteome (Fig. 1, G and I, and data S2). Pathway analysis revealed the presence of mitochondrial matrix proteins (galactokinase, GALK1; pyruvate dehydrogenase component, PDHX; fumarate hydratase, FH) and nucleoid and mitochondrial inner membrane proteins (DNAJC11; prohibitin 2, PHB2; OXA1L), pointing to a rupture of the mitochondrial outer membrane and consequent biotinylation of mitochondrial matrix components. In addition, we also detected proteins related to coated vesicles, such as the retromer subunit VPS29 and RAB10, together with the lysosomal proteins MYO1B, MYO6, ATP6V0D1, and cathepsin D (CTSD). Thus, mtDNA stress changes the proximity proteome of mitochondria toward lysosomal recruitment to facilitate the degradation of mitochondrial components.

### The retromer facilitates mitochondrial fragmentation upon mtDNA replication stress

The retromer is a trimeric protein complex formed by three components: VPS35, VPS29, and VPS26, mainly the isoform VPS26A. The main function of this complex is to control vesicle trafficking among endosomes and lysosomes and promote their maturation and degradative activity (*21*). However, upon certain conditions, the retromer and primarily its core component, VPS35, participates in mitochondrial quality control. In an oxidative environment, the retromer generates mitochondrial vesicles directed to the peroxisomes (*22*). Upon mtDNA replication stress, the mitochondrial genome transfers to retromer foci (*13*). How this translocation occurs is not clear.

**Fig. 2.**
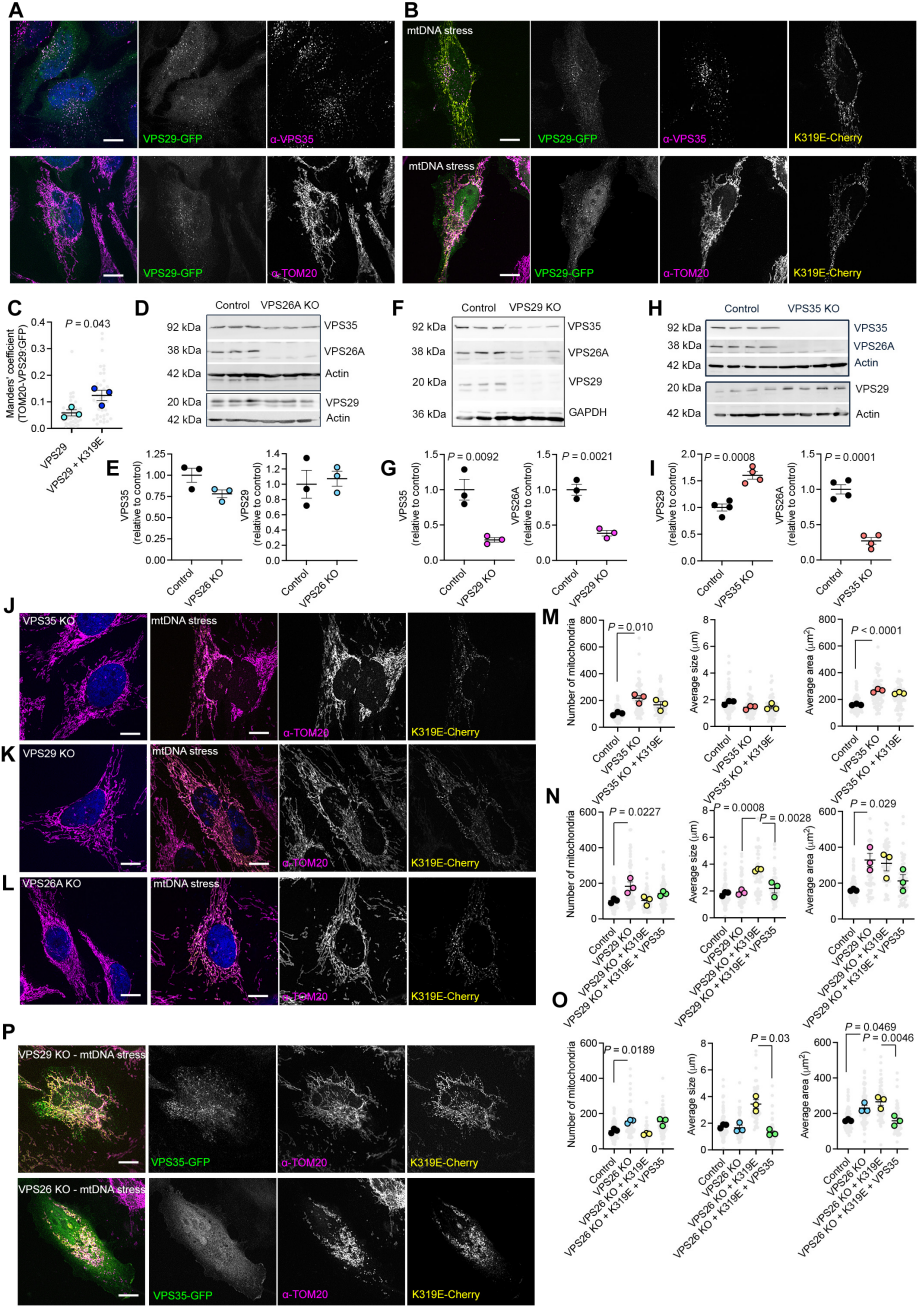
The retromer participates in the response to mtDNA replication stress by adapting the mitochondrial morphology. (**A** and **B**) Immunostaining of HeLa cells expressing VPS29-GFP and (B) TWNK^K319E^-mCherry labeled with α-VPS35 and α-TOM20. (**C**) Manders’ correlation coefficient between TOM20 and VPS29. *n* = 3, 10 images per replicate. (**D** to **I**) Western blot analysis and quantification of retromer components upon [(D) and (E)] VPS26A, [(F) and (G)] VPS29, or [(H) and (I)] *VPS35* KO. α-GAPDH or α-actin was used as a loading control. GAPDH, glyceraldehyde-3-phosphate dehydrogenase. (**J** to **L**) α-TOM20 immunostaining in (J) *VPS35*, (K) *VPS29*, and (L) *VPS26* KO, in the steady state and expressing TWNK^K319E^-mCherry. (**M** to **O**) Quantification of mitochondrial morphology parameters. *n* = 3, >20 cells per replicate. (**P**) α-TOM20 immunostaining in *VPS29* and *VPS26A* KO expressing TWNK^K319E^-mCherry and VPS35-GFP. *P* values were calculated using Student’s *t* test [(C), (E), (G), and (I)] and one-way ANOVA [(M) to (O)]. Scale bars, 10 μm. Data are presented as means ± SEM.

To investigate how the retromer licenses mitochondrial quality control pathways during mtDNA stress, we first analyzed its impact on mitochondrial dynamics. As described, at a steady state, expression of the retromer component VPS29-green fluorescent protein (GFP) formed cytosolic foci colocalizing within the other retromer components, VPS35 and VPS26A, but not mitochondria (Fig. 2A and fig. S2A). Immunostaining with other vesicle proteins showed partial colocalization with both early (RAB5) and late endosomes (LAMP1) (fig. S2A). TWNK^K319E^ did not modify the subcellular pattern of the retromer within the vesicular network (fig. S2B). Nevertheless, we noticed higher colocalization of VPS29 with mitochondria (Fig. 2, B and C). Expression of either VPS29-GFP, VPS35-GFP, or TWNK^K319E^-mCherry alone had no major effect on the mitochondrial network morphology (fig. S2, C, D, and F). Similarly, cells coexpressing both VPS29–GFP and TWNK^K319E^-mCherry showed no differences (fig. S2, E and F). However, cells coexpressing VPS35-GFP and TWNK^K319E^-mCherry showed robust mitochondrial fragmentation, depicted by an increased number of mitochondria and reduced average size (fig. S2, E and F).

Further, we generated CRISPR-Cas9 HeLa knockout (KO) cells for each retromer subunit. The KO of *VPS26A* did not affect other retromer subunits (Fig. 2, D and E). On the contrary, *VPS29* KO decreased the steady-state level of VPS35 and VPS26A (Fig. 2, F and G). *VPS35* KO led to an up-regulation of VPS29 and decreased VPS26A (Fig. 2, H and I). Then, we analyzed the effect of the KO on the mitochondrial morphology (Fig. 2, J to L). In the steady state, all three KO cell lines presented an increased number of mitochondria and mitochondrial area (Fig. 2, M to O). Opposite to what we observed in wild-type (WT) cells, the expression of TWNK^K319E^ led to mitochondrial elongation in *VPS26* and *VPS29* KO, whereas no effect was observed in *VPS35* KO cells (Fig. 2, J to O). We wondered whether the lack of mitochondrial fission induced by mtDNA stress and observed upon the KO of any of the three retromer subunits was due to the reduced levels of VPS35 in all KO models (Fig. 2, D to I). Consistently, the reexpression of *VPS35* in *VPS29* and *VPS26* KO restored mitochondrial fragmentation (Fig. 2, M, O, and P).

These data collectively revealed that the retromer is necessary for the mitochondrial response to mtDNA replication stress, and its core component VPS35, independently of its accessory subunits, retains the ability to fragment mitochondria.

### The retromer facilitates the release of mitochondrial matrix components during mitochondrial stress

One proposed mechanism for retromer-dependent mitochondrial quality control is the formation of MDVs (*22*). MDVs are intermediate structures formed upon different types of stresses and containing selective cargo. Up to now, many types of vesicles derived from the mitochondria have been described. MDVs contain either one of both mitochondrial membranes and are enriched for specific cargo (*23*). The involvement of the mitochondrial fission factor Dynamin-Related Protein 1 (DRP1) seems to depend on the type of vesicle (*24*, *25*). MDVs are, however, independent of autophagosome-related proteins (*26*).

**Fig. 3.**
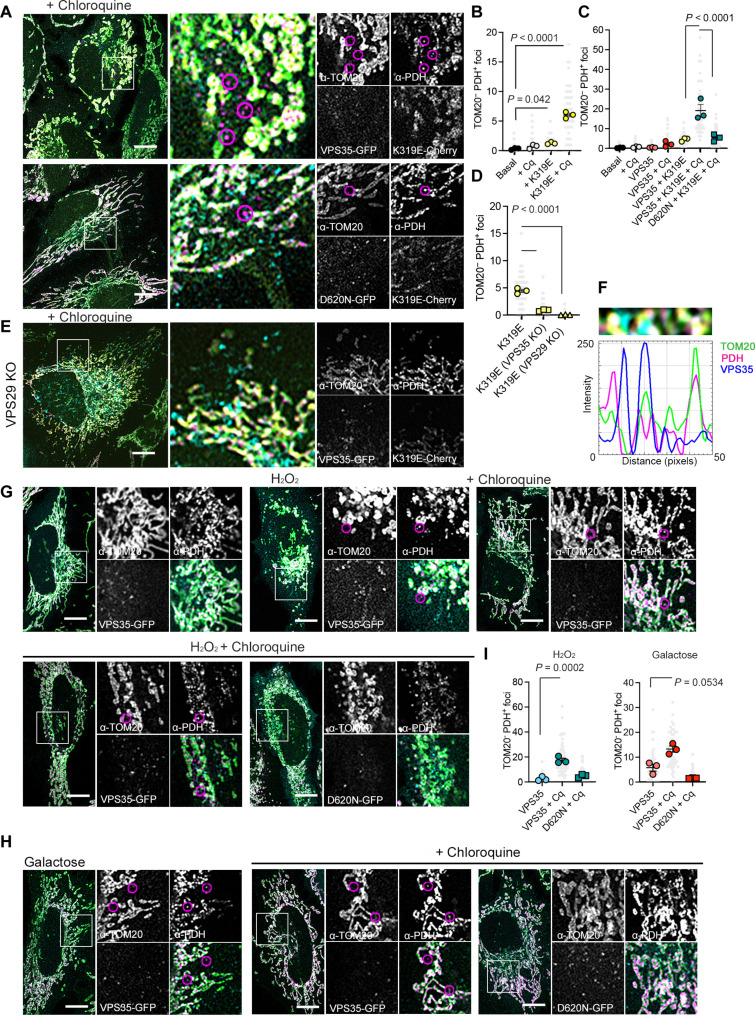
mtDNA replication stress and oxidation initiate the release of mitochondrial components. (**A**) HeLa cells expressing VPS35-GFP and TWNK^K319E^-mCherry and labeled with the mitochondrial outer membrane marker α-TOM20 and mitochondrial matrix α-PDH. (**B** to **D**) Quantification of α-TOM20− and α-PDH+ foci [(B) and (C)] in control cells expressing the indicated plasmids and (D) in retromer deficient cells. *n* = 3, >15 cells per replicate. (**E**) Immunostaining of VPS29 KO cells expressing TWNK^K319E^-mCherry and labeled with α-TOM20− and α-PDH+ foci. (**F**) Fluorescence profile of 50-pixel line. The selected area is highlighted above the graph. (**G** and **H**) Immunostaining of α-TOM20 and α-PDH in cells treated with (G) 200 μM H_2_O_2_ 4 hours or (H) grown in galactose medium overnight. (**I**) Quantification of α-TOM20− and α-PDH+ foci in H_2_O_2_ and galactose-treated cells. Where indicated, cells were treated with 10 μM chloroquine 4 hours before fixation to block lysosomal function (*n* = 3, >15 cells per replicate). TOM20−/PDH+ foci are encircled in magenta circles. *P* values were calculated using one-way ANOVA with Tukey correction for multiple comparisons. Scale bars, 10 μm. Data are presented as means ± SEM.

Thus, we first examined the presence of vesicle-like structures induced by the retromer and mtDNA replication stress. As an internal control, we included the Parkinson’s disease (PD)–associated mutation VPS35^D620N^, known to restrict cargo selection and mitochondrial clearance (*27*, *28*). Because the mitochondrial genome resides in the mitochondrial matrix, we tested for the presence of vesicles carrying pyruvate dehydrogenase (PDH) and lacking the outer membrane protein TOM20 (*29*). In the steady state, VPS35 did not colocalize with the mitochondrial markers PDH or TOM20 (fig. S3A). Expression of TWNK^K319E^-mCherry induced the formation of sparse TOM20−/PDH+ foci, which accumulated upon lysosomal inhibition with chloroquine (Fig. 3, A and B). Very few vesicles were observed upon chloroquine treatment alone or expression of VPS35-GFP alone (Fig. S3B and C), suggesting that they rarely formed in a steady state. The coexpression of TWNK^K319E^ and VPS35 increased the accumulation of TOM20−/PDH+ foci (Fig. 3C), which colocalize with the late endosomal/lysosomal marker LAMP1 (fig. S3, D and E).

Cells expressing VPS35^D620N^-GFP or VPS35 KO cells showed reduced TOM20−/PDH+ vesicles (Fig. 3, A, C, and D). On the other hand, the expression of VPS29 did not induce the formation of any type of mitochondrial vesicles (fig. S3F), suggesting again that VPS35 retains the mitochondrial fission activity. Moreover, TWNK^K319E^ did not induce any TOM20−/PDH+ vesicles without VPS29 (Fig. 3D), although VPS35 was still recruited to the mitochondria (Fig. 3, E and F).

Next, we asked whether the formation of these vesicular structures relies on the MDVs machinery, specifically Miro1 (*RHOT1*) and DRP1 (*6*). To test this, we generated Miro1 KO HeLa cells and expressed TWNK^K319E^-mCherry along with VPS35-GFP (fig. S3, G to I). Although we detected sparse TOM20−/PDH+ foci, their number remained unchanged upon mtDNA replication stress (fig. S3F). Similarly, *DRP1* KO HeLa cells showed minimal independent TOM20−/PDH+ foci (fig. S3, G to I). Notably, *DRP1* KO cells maintained an elongated mitochondrial network, even upon TWNK^K319E^ expression (fig. S3J). Likewise, Miro1 KO cells displayed a tubulated network, which became aggregated under mtDNA replication stress (fig. S3J).

Furthermore, we asked whether the elimination of mitochondrial content mediated by the retromer also occurred when interfering with mitochondrial function by other means. Given that mtDNA replication stress causes oxidative damage (*13*) and this is one of the most common forms for natural acquisition of mtDNA mutations (*30*), we treated our cells with a sublethal concentration of H_2_O_2_. In parallel, we also grew the cells in galactose, a condition that supports mitochondrial metabolism and oxidation (*31*). Thus, we observed the formation of TOM20−/PDH+ foci enriched upon the inhibition of the lysosomal function with chloroquine (Fig. 3, G to I). Again, these particles were not detectable upon the expression of the PD-associated mutant VPS35^D620N^ (Fig. 3, G to I).

Recently, a mechanism involved in the elimination of the mitochondrial inner membrane, and occasionally mtDNA, was described to follow vesicle formation through VDAC1 pores (*11*). However, upon mtDNA replication stress, immunostaining of the inner mitochondrial membrane subunit of the complex V ATPase (adenosine triphosphatase), ATP5A, showed no foci independent of the outer membrane protein TOM20 (fig. S3K).

Together, these findings confirm that the downstream response to the mtDNA replication stress includes mitochondrial vesiculation controlled by Miro1 and DRP1, independent of vesicles derived from the inner membrane (VDIMs) (*11*). mtDNA replication stress follows the activation of lysosomal-dependent quality control mechanisms for the mitochondrial matrix that is related to vesicle formation, resembling the MDV pathway.

### mtDNA ejection during mtDNA stress occurs directly to vesicles from the endosomal system

**Fig. 4.**
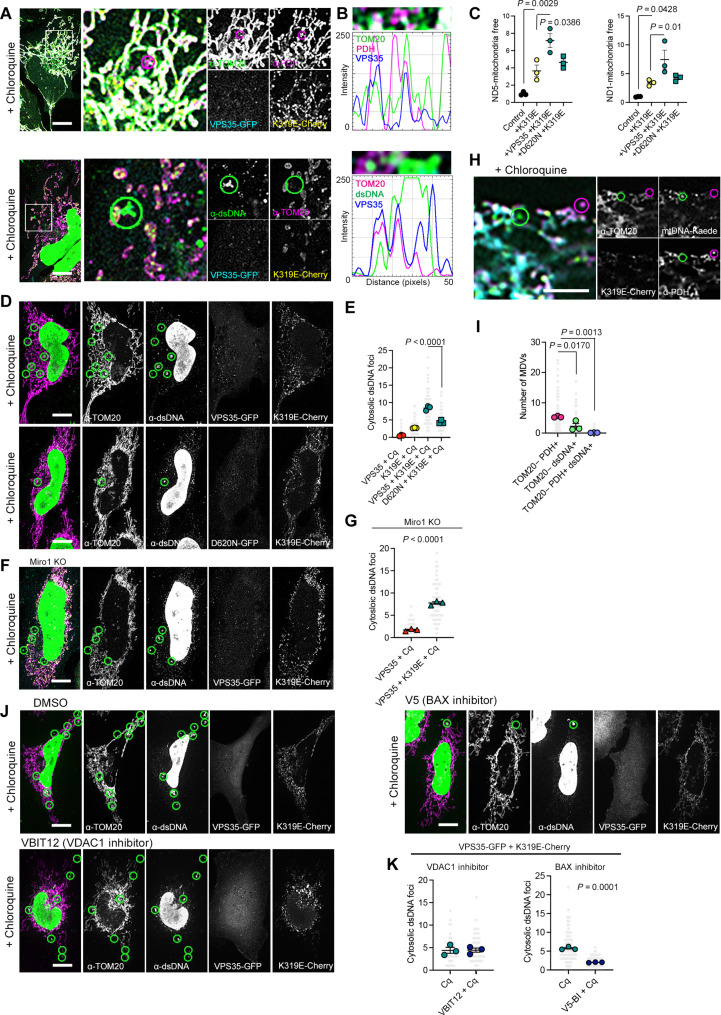
mtDNA replication stress triggers Bax-dependent mtDNA release. (**A**) HeLa cells expressing TWNK^K319E^-mCherry and VPS35-GFP labeled with α-TOM20 and α-dsDNA or α-PDH. (**B**) Fluorescence profile of 50-pixel line labeled in (A). (**C**) qPCR quantification of *ND1* and *ND5* mtDNA genes in mitochondria-free cytosolic fractions (*n* = 3). (**D** to **G**) Analysis and quantification of cytosolic dsDNA foci upon mtDNA replication stress by immunostaining with α-TOM20 and α-dsDNA in [(D) and (E)] VPS35-GFP expressing cells and [(F) and (G)] Miro1 KO cells. (*n* = 3, >15 cells per replicate). (**H**) HeLa cells expressing mtDNA-Kaede and TWNK^K319E^-mCherry labeled with α-TOM20 and α-PDH. (**I**) Quantification of TOM20- Vesicles containing PDH, dsDNA or both (*n* = 3, >20 cells per replicate). (**J** and **K**) Analysis and quantification of the involvement of mitochondrial membrane pores in mtDNA release. Where indicated, 24 hours after transfection, cells were further treated 4 hours with 10 μM chloroquine, 25 μM VBIT-12 for VDAC1 inhibition, or 100 μM V5 peptide for the inhibition of BAX oligomerization (*n* = 3, >15 cells per replicate). TOM20−/PDH+ foci are encircled by magenta circles. TOM20−/dsDNA^+^ and TOM20−/mtDNA-Kaede^+^ foci are encircled by magenta circles. DMSO, dimethyl sulfoxide. *P* values were calculated using one-way ANOVA with Tukey correction for multiple comparisons [(C), (E), and (I)] and Student’s *t* test [(G) and (K)]. Scale bars, 10 μm, except for (H), 5 μm. Data are presented as means ± SEM.

We sought to investigate whether the elimination of the mitochondrial genome follows the same path observed for mitochondrial matrix components. We found VPS35-GFP in the exit sites of both PDH and the mtDNA (Fig. 4, A and B). To explore the role of the retromer in the extraction of mtDNA, we generated cells stably expressing VPS35-V5. Quantitative polymerase chain reaction (qPCR) analysis of mtDNA genes in mitochondria-free fractions showed increased *MT-ND1* and *MT-ND5* in cells with mtDNA replication stress (Fig. 4C and fig. S4A). VPS35 overexpression further increased extraction, whereas no changes were observed in cells with the dominant negative VPS35^D620N^. Consistently, coexpression of TWNK^K319E^ and VPS35 increased the number of cytosolic double-stranded DNA (dsDNA) foci, which were further enriched upon lysosomal inhibition with chloroquine (Fig. 4, D and E, and fig. S4, B to D). In contrast, cells expressing VPS35^D620N^ exhibited less extra-mitochondrial dsDNA (Fig. 4, D and E). Unexpectedly, Miro1 KO cells also displayed nonmitochondrial dsDNA foci (Fig. 4, F and G; Fig. S4E). Notably, MDV analysis in cells transfected with mtDNA-Kaede (fig. S4F) showed either TOM20−/PDH+ foci or TOM20−/mtDNA+, and almost none TOM20−/PDH+/dsDNA+ vesicles (Fig. 4, H and I, and fig. S4G). These data suggest that, although mitochondrial matrix MDVs and mtDNA release are activated under mtDNA replication stress, mtDNA release occurs independently of the vesiculation of the mitochondrial matrix.

Thus, we investigated whether mitochondrial pores contribute to the mtDNA release. Cells treated with the VDAC1-specific inhibitor VBIT-12 (*32*) in combination with chloroquine showed no changes in the number of cytosolic dsDNA foci (Fig. 4, J and K). In contrast, treatment with the BAX inhibitor peptide V5 (*33*) showed a significantly reduced number of nonmitochondrial dsDNA foci (Fig. 4, J and K).

**Fig. 5.**
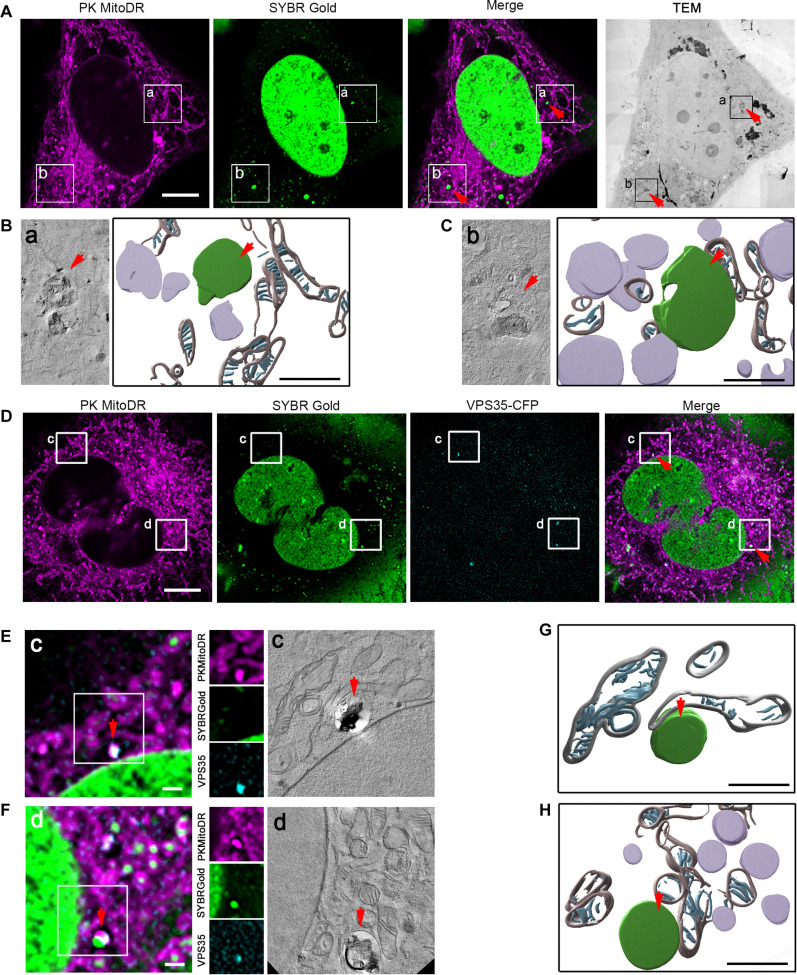
mtDNA release occurs through direct transfer to a recycling organelle. (**A**) CLEM of cells expressing TWNK^K319E^-mCherry and labeled with SYBR Gold and PK MitoDeep Red. Boxes labeled with (a) and (b) indicate the areas used for CLEM. (**B** and **C**) Volumetric reconstitution of electron tomographies of cytosolic mtDNA areas. The organelle containing dsDNA is shown in green, other recycling organelles in lilac, mitochondrial outer membrane in gray, and mitochondrial cristae in cyan. (**D**) TWNK^K319E^-mCherry cell further expressing VPS35-CFP. Boxes labeled with (c) and (d) indicate the areas used for CLEM. (**E** and **F**) Correlation of VPS35-CFP foci and (F) mtDNA exit site. (**G** and **H**) Volumetric reconstitution of electron tomographies in VPS35-CFP cells. In all cases, cells were treated with 10 μM chloroquine for 4 hours. For CLEM, all dyes were loaded for 1 hour before fixation. Scale bars, 500 nm for reconstituted tomograms [(B), (C), (G), and (H)], 1 μm [(E) and (F)], and 10 μm [(A) and (D)].

Our data challenge the notion of vesicle-mediated mtDNA release, suggesting that, upon mtDNA replication stress, the mitochondrial genome may be released directly into the cytoplasm. To visualize the cytosolic dsDNA foci in high resolution and gain information about the cellular context, we performed CLEM and combined it with electron tomography. We stained chloroquine-treated cells with SYBR Gold for DNA and PK Mito Deep Red for mitochondria (fig. S5A). In contrast to control cells, cells expressing TWNK^K319E^-mCherry showed cytosolic DNA foci (Fig. 5A and fig. S5B). These cells presented reduced mitochondrial size and fewer cristae per mitochondria (fig. S5, C to E). The correlation of confocal images with transmission electron microscopy and electron tomography showed that areas containing cytosolic dsDNA were enriched in recycling organelles at different stages of maturation (Fig. 5, B and C; fig. S4, F and G; and movie S1). None of the cells analyzed with CLEM (*n* = 6) showed DNA inside a small independent free cytosolic vesicle or membrane-free in the cytoplasm. Next, we performed the same approach in cells expressing TWNK^K319E^-mCherry and VPS35–cyan fluorescent protein (CFP) (Fig. 5D and fig. S4H). Correlation experiments showed the presence of VPS35-CFP in the same type of recycling organelle containing electron-dense material and in close apposition with mitochondria (Fig. 5, E and G, and movie S2). Electron tomography reconstitution of one mtDNA exit site confirmed the transfer of mtDNA to one of these nearby vesicles also labeled with VPS35-CFP (Fig. 5, F and H, and movie S3). Consistently, immunostaining of TWNK^K319E^-mCherry cells showed an increased number of dsDNA in contact with the lysosomal/late endosomal marker LAMP1 (fig. S5, I and J).

### Active lysosomes facilitate mtDNA turnover

One of the main functions of the retromer complex is the maturation of early endosomes toward late endosomes/lysosomes. In HeLa cells, the retromer promotes the processing of lysosomal hydrolases, influencing lysosomal morphology and function (*34*). Lysosomes are highly dynamic organelles that move fast in cells, adapting the degradative capacity of the cells to cellular needs (*35*). The positioning of lysosomes within the cell affects their activity and interactions with other organelles (*36*). Perinuclear lysosomes are typically involved in degradation, whereas those in the periphery function in the endocytic processes. Rab guanosine triphosphatases (GTPases) coordinate with the retromer complex to regulate lysosomal positioning and trafficking (*37*).

One of the proteins that we identified by proximity proteomics was RAB10 (Fig. 1I). Upon different stimuli, RAB10 influences the distribution of lysosomes between the perinuclear region and the cell periphery, thereby affecting cellular processes like autophagy and lysosomal degradation (*38*). Upon mitochondria depolarization, RAB10 binds to the autophagy receptor optineurin and promotes its accumulation, thereby facilitating mitophagy (*39*).

To determine how RAB10-lysosomes participate in the downstream response to mtDNA stress, we used the expression of three different alleles, RAB10 WT, the gain-of-function allele RAB10^Q68L^, and the dominant negative RAB10^T23N^. The activity of RAB10 is influenced by its binding status to GTP (guanosine triphosphate) (*40*). Although the WT allele can shift from an active to an inactive form, the gain-of-function always remains active, in contrast to the dominant negative mutation, which always remains inactive. Therefore, the changes induced by RAB10 are expected to be enhanced for the RAB10^Q68L^ allele.

**Fig. 6.**
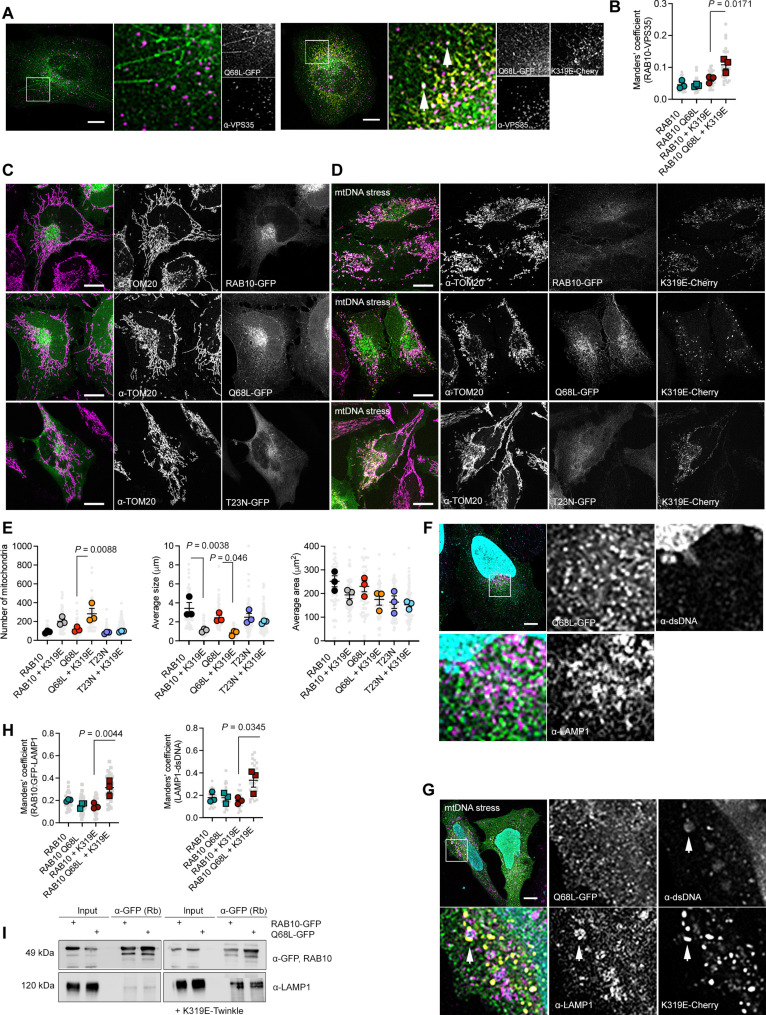
The small GTPase RAB10 promotes mitochondrial fragmentation and mtDNA degradation in lysosomes. (**A**) Immunostaining of HeLa cells expressing the constitutive active protein RAB10^Q68L^-GFP labeled with α-VPS35. (**B**) Manders’ correlation coefficient between RAB10 and VPS35. (**C** and **D**) Confocal images of cells expressing WT RAB10-GFP, constitutive active RAB10^Q68L^-GFP, dominant negative RAB10^T23N^-GFP, in the steady state, and (D) expressing TWNK^K319E^-mCherry, labeled with α-TOM20. (**E**) Quantification of the mitochondrial morphology in RAB10 expressing cells (*n* = 3, >20 cells per replicate). (**F** and **G**) Cells expressing RAB10^Q68L^-GFP and (G) TWNK^K319E^-mCherry labeled with α-LAMP1 and α-dsDNA. Arrows depict RAB10-LAMP1-dsDNA foci. (**H**) Manders’ correlation coefficient between RAB10-GFP and LAMP1 and LAMP1 and dsDNA (*n* = 3, 10 images per replicate). (**I**) RAB10-GFP coimmunoprecipitation in the steady state and cells expressing TWNK^K319E^-mCherry with the lysosomal protein LAMP1. *P* values were calculated using one-way ANOVA with Tukey correction for multiple comparisons. Scale bars, 10 μm. Data are presented as means ± SEM.

Consistently, mtDNA replication stress increased the association of RAB10^Q68L^-GFP with the retromer, pointing to the activation of a lysosome-dependent path to degradation (Fig. 6, A and B). Quantitative analysis of mitochondrial morphology showed that, upon mtDNA replication stress, cells expressing WT RAB10 or the gain-of-function allele RAB10^Q68L^ present an increased number of mitochondria and reduced average size, more prominent for the gain-of-function allele. However, no changes were detectable in cells expressing the dominant negative variant (Fig. 6, C to E).

Then, we investigated the subcellular location of RAB10 upon mtDNA stress. In the steady state, RAB10 partially localized with late endosomes, but no dsDNA could be detected in these particles (Fig. 6F). Upon mtDNA replication stress, RAB10 accumulated in LAMP1-positive late endosomes together with extra-mitochondrial dsDNA (Fig. 6G). In agreement, colocalization between RAB10 and LAMP1, as well as between LAMP1 and dsDNA, was enhanced (Fig. 6H). Pull-down experiments with RAB10-GFP confirmed a strong association with LAMP1 upon mtDNA stress (Fig. 6I). In summary, these data confirm that the downstream response associated with mtDNA replication stress is regulated by RAB10-active lysosomes.

### Generation of long mtDNA deletion in *Drosophila* larval epidermis

**Fig. 7.**
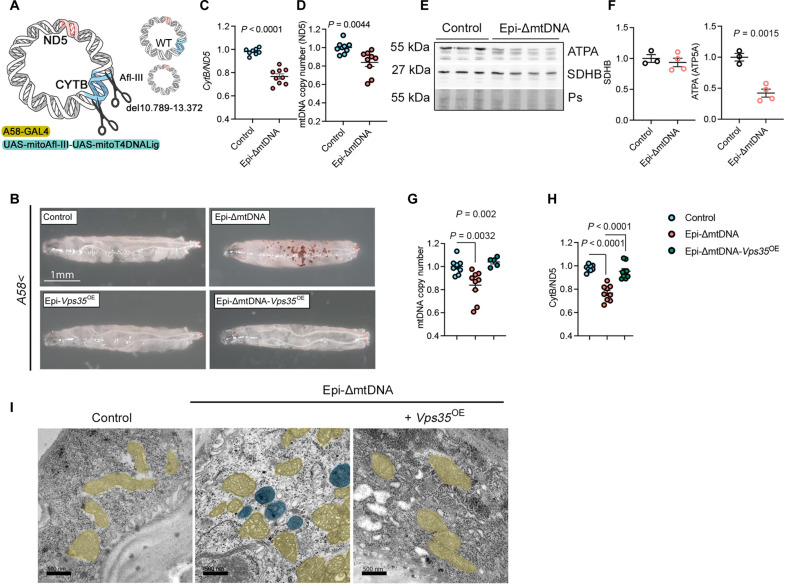
VPS35 overexpression recovers mtDNA homoplasmy in *Drosophila*. (**A**) Schematic representation of the approach to generate mtDNA deletion covering 2564 bp in larval epidermis. The restriction enzyme Afl III and the T4-DNA ligase directed to mitochondria were both expressed under the UAS promotor. A-58 Gal4 was used to drive the expression of the transgenes in the larval epidermis. WT, wild-type mtDNA. Illustration from NIAID NIH BIOART Source. (**B**) Bright-field images of transgenic L3 larvae used in this study. (**C**) *CytB/ND5* ratio showing mtDNA heteroplasmy obtained by qPCR from total DNA extracts of L3 larvae (Control, *n* = 8; Epi-ΔmtDNA, *n* = 9). (**D**) mtDNA copy number quantification using *ND5* gene and *TUBB* as a mitochondrial and nuclear gene, respectively (*n* = 9). (**E** and **F**) Western blot analysis (E) and quantification (F) of total protein extracts of L3 larvae for SDHB and ATPA. Ponceau S (Ps) was used as a loading control (Control, *n* = 3; Epi-ΔmtDNA, *n* = 4). (**G** and **H**) mtDNA copy number (G) and mtDNA heteroplasmy quantification (H) upon genetic manipulations (Control, *n* = 9; Epi-ΔmtDNA, *n* = 9; Epi-ΔmtDNA-*Vps35*^OE^, *n* = 9). (**I**) Electron microscopy images of larval epidermis. Mitochondria were pseudocolored in yellow and lysosomes in blue (dark content). Control refers to the *A58Gal4*/+ genotype (B) and specific UAS control in other quantifications. *P* values were calculated using the Student’s *t* test [(C), (D), and (F)] and one-way ANOVA with Tukey correction for multiple comparisons [(G) and (H)]. Scale bars, 1 mm (B) and 500 nm (I). Data are presented as means ± SEM.

Our findings suggest that the retromer enhances specific quality control mechanisms for the mtDNA. We hypothesized that overexpression of VPS35 might also be beneficial in vivo, by reducing the mitochondrial burden associated with mtDNA mutations. To investigate our hypothesis, we used a well-established Gal4/upstream activated sequence (UAS) *Drosophila* model based on nucleases (*41*). *Drosophila* mtDNA harbors two target sites for the restriction enzyme Afl III. Coexpression of mitochondria-targeted Afl III (mitoAfl III) and the DNA ligase from phage T4 (mitoT4lig) (Fig. 7A; *UAS-mitoAfl III*, *UAS-mitoLigase*) results in the generation of a 2564–base pair (bp) deletion in the mtDNA, including the *CytB* gene (mtDNA^Δ10.789-13.372^).

Simultaneous induction of both UAS constructs (*UAS-mitoAfl III*, *UAS-mitoLigase*) by most tested Gal4 drivers (neurons, epithelial, muscles, or fat bodies) leads to embryonic or early larval lethality (*41*). Consistently, upon induction mediated by the larval epidermal driver A58 at 25°C (*A58-Gal4* > *UAS-mitoAflIII*, *UAS-mitoLigase*; from now on Epi-ΔmtDNA), only a few larvae could survive to the pupal stage. To overcome the lethality, we maintained the crosses at 18°C. At this temperature, the Gal4 activity is at its lowest levels, leading to larval survival till the pupal stage (*42*). At this temperature, we noticed the presence of restricted chromogenic foci but larvae could reach pupation, although only ~1% of pupae emerged (Fig. 7B). Hence, this temperature and L3 larvae were selected for further experiments.

Expression of mitoAfl III was confirmed to peak in the early L3 instar stage and decay in the mid-stage (L3-E versus L3-M; fig. S6A). Conventional PCR using oligos flanking mtDNA^Δ10.789-13.372^ confirmed the presence of the mtDNA deletion in Epi-ΔmtDNA (fig. S6B). Accordingly, the *CytB*/*ND5* ratio was reduced (Fig. 7C) and the mtDNA copy number decreased, to a bigger extent when *CytB*, included in the mtDNA^Δ10.789-13.372^ deletion, was used for mtDNA copy number quantification (Fig. 7D and fig. S6C). Western blot analysis from total protein extracts showed equal levels of the mitochondrial complex II subunit succinate dehydrogenase B (SDHB) and depletion of complex V subunit ATPA (Fig. 7, E and F). Given that mitochondrial complex II is encoded by nuclear genes and complex V by both nuclear and mitochondrial genomes, this result confirmed that mtDNA depletion was not related to reduced mitochondrial mass but due to a lower number of mtDNA copies. Thus, the coexpression of mitoAfl III and mitoT4lig in the epidermis of *Drosophila* larvae efficiently deletes mtDNA and increases mtDNA heteroplasmy.

### VPS35 promotes mtDNA clearance in vivo

To test whether *Vps35* elevation supports mtDNA clearance in vivo, we overexpressed *Vps35* in the larval epidermis by crossing the UAS-Vps35 line with the A58-Gal4 driver (Epi-*Vps35*^OE^). Epi-*Vps35*^OE^ larvae did not exhibit macroscopic abnormalities with no observable morphological or developmental defects (Fig. 7B). qPCR quantification of total RNA extracts revealed a ninefold increase in *Vps35* mRNA (Fig. S6D). In addition, Epi-*Vps35*^OE^ larvae had a moderate but significant increase in mtDNA copy number (fig. S6E). Next, we generated Epi-ΔmtDNA*-Vps35*^OE^. In this background, *Vps35* mRNA levels were increased fivefold on average compared to *Epi-ΔmtDNA* (fig. S6F). We did not observe major macroscopic and morphological abnormalities in larvae and the chromogenic foci initially observed in Epi-ΔmtDNA larvae disappeared (Fig. 7B). Quantification of mtDNA copy number and *CytB/ND5* ratio showed normalized mtDNA copies in Epi-ΔmtDNA-*Vps35*^OE^ compared to control larvae (Fig. 7, G and H). Moreover, Western blot data confirmed higher levels of the complex V protein ATPA and no changes in the nuclear-encoded complex II protein SDHB (fig. S6, G and H).

Last, we investigated changes in the mitochondria ultrastructure triggered by these genetic modulations. Electron microscopy preparation of the larvae epidermis showed cristae aberrations in the epidermis of Epi-ΔmtDNA animals (Fig. 7I). In addition, we noticed the accumulation of lysosomes and multivesicular bodies intercalated with mitochondria (Fig. 6I, blue pseudocolored). Epi-*Vps35*^OE^ in Epi-ΔmtDNA background showed a complete recovery of mitochondrial ultrastructure similar to control larvae.

### Elevated *Vps35* reverses transcriptome profile in the epidermis of ΔmtDNA larvae

**Fig. 8.**
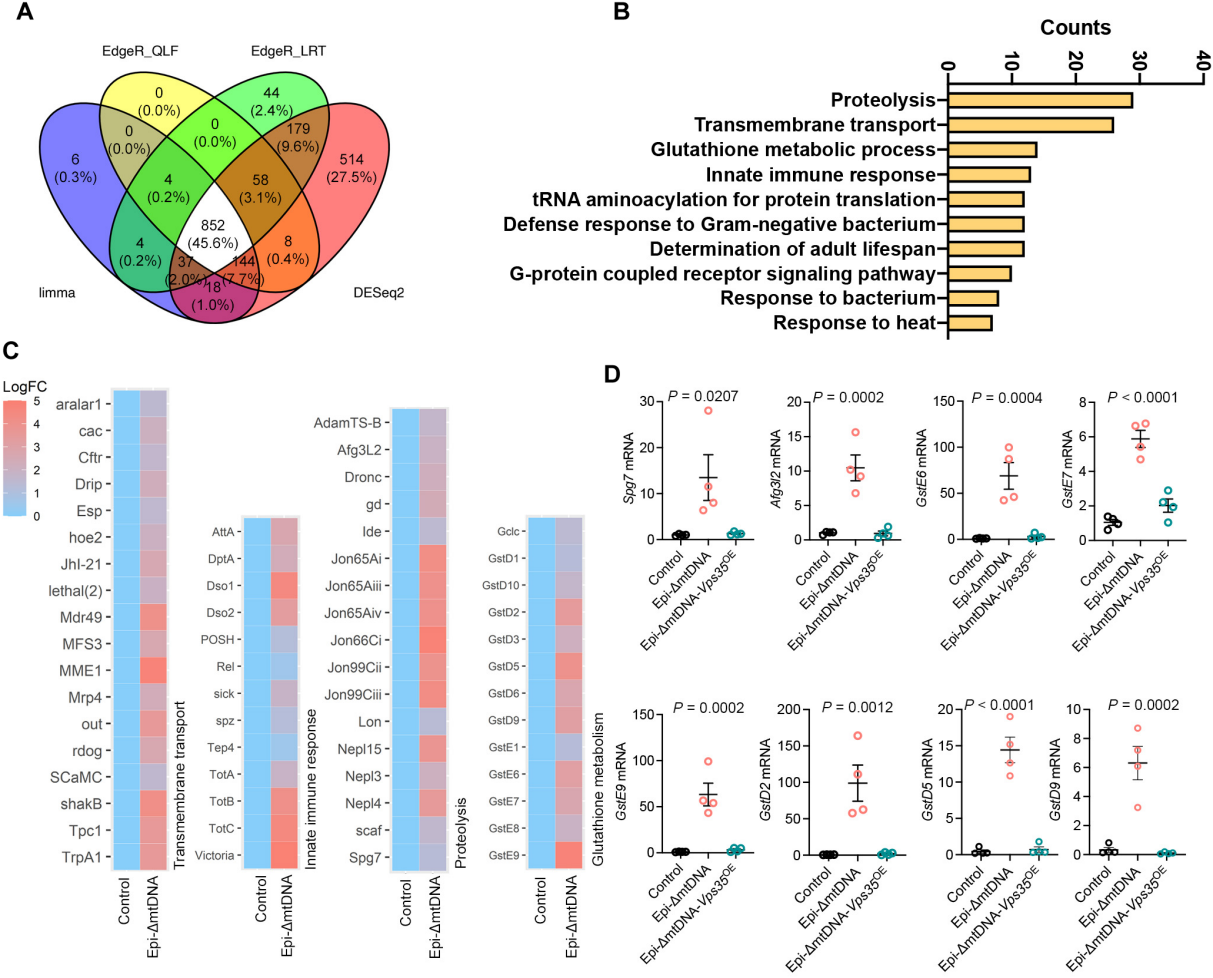
VPS35 expression restores mitochondrial defects associated with ΔmtDNA. (**A**) Venn diagram for genes differentially expressed (FDR of 5%) between control and Epi-ΔmtDNA shared through four differential expression methods (Limma-voom, EdgeR-QLF, EdgeR_LRT, and DESeq2) (*n* = 4 per genotype). (**B**) DAVID pathway enrichment analysis for genes differentially expressed. (**C**) Heatmap for log FC showing gene identities for the top four categories of differentially enriched pathways. (**D**) mRNA quantification of selected genes in Epi-ΔmtDNA and Epi-ΔmtDNA-*Vps35*^OE^ larvae (*n* = 4 per genotype). Control refers to the *A58-Gal4*/+ genotype. *P* values were calculated using one-way ANOVA with Tukey correction for multiple comparisons. Data are presented as means ± SEM.

For an unbiased and genome-wide overview of different pathways activated in ΔmtDNA epidermis, we conducted a transcriptome analysis in isolated epidermis from L3 ΔmtDNA larvae (fig. S7, A and B). As expected, due to the presence of the deletion, mtDNA^Δ10.789-13.372^, mitochondrial mRNAs for *CytB*, *ND1*, and *lrRNA*, genes included in the deletion, were strongly reduced (fig. S7C). Then, we performed differential expression analysis using four independent bioinformatic methods (EdgeR-QFL, EdgeR-LRT, limma-voom, and DESeq2) and identified 541 genes with significant up-regulation, whereas 311 genes were down-regulated (Fig. 8A and data S3). Pathway analysis with DAVID (Database for Annotation, Visualization, and Integrated Discovery) and Gene Set Enrichment Analysis (GSEA) with Gene Ontology (GO) pathways uncovered up-regulation of proteolysis, innate immune response, and redox control routes (Fig. 8, B and C; fig. S7, D and E; and data S3). To further validate these data, we selected some of the up-regulated targets and performed qPCR analysis in independent RNA isolates. We found consistent enrichment of the glutathione *S*-transferase genes *GstD2*, *GstD5*, *GstD9*, *GstE5*, *GstE7*, and *Gst9*, along with the mitochondrial proteases *Spg7* and *Afg3l2* (Fig. 8D). Epi-*Vps35^OE^* in the background of Epi-ΔmtDNA larvae (fig. S6I) reversed all mRNA levels for the selected targets to normal (Fig. 8D).

### VPS35 elevation supports lysosomal function without affecting macroautophagy

To understand the molecular mechanisms by which VPS35 restores the mitochondrial function, we turned once again to human cells. Given that the retromer participates in lysosomal homeostasis, we hypothesized that higher levels of VPS35 would improve the cellular degradation pathways. LysoTracker staining in cells expressing VPS35-V5 and TWNK^K319E^-mCherry showed a higher number of acidic particles than in control cells (fig. S8, A and B). In line with this, in *VPS35* KO cells, the number of acidic foci was down-regulated (fig. S8, A and B).

Next, we wondered whether these effects on lysosomal pH related to VPS35 overexpression were also linked to changes in macroautophagy. Western blot analysis of autophagy markers showed increased levels of LC3B-II and p62 at the steady state in cells overexpressing VPS35-V5 (fig. S8, C and D). Autophagy flux experiments in chloroquine-treated cells revealed no differences for LC3B-II accumulation, but VPS35-overexpressing cells showed a subtle increase in the adaptor protein p62 and VPS35 itself. At the same time, the lysosomal marker LAMP1 was highly enriched in VPS35 cells (fig. S8, E and F).

Confocal microscopy showed an increased association of mitochondria with late endosomes LAMP1 in VPS35 overexpressing cells (fig. S8, G and H). We wondered whether this increased association translated into an increase in the macroautophagy of the complete mitochondria. Thus, we transduced our cells with mitoKeima, a fluorescence reporter with a pH-dependent fluorescence excitation profile. In the mitochondria, mitoKeima has an excitation peak at 448 nm, whereas in the lysosomes, due to the acidic pH, the excitation peak shifts to 561 nm. In both cases, the fluorescence emission remains at 610 nm. The ratiometric analysis of mitoKeima fluorescence emission, when excited sequentially at 586 nm/440 nm, showed a decrease in bulk mitophagy for cells overexpressing VPS35 (fig. S8I).

All these data together confirm that VPS35 elevation does not affect the general autophagy pathways, including bulk mitophagy. However, it stimulates the lysosomal function upon stress, accelerating the piecemeal removal of mitochondrial components and facilitating recovery upon mtDNA damage.

## DISCUSSION

Mitochondrial quality control serves as a salvage pathway to eliminate ill-functioning mitochondria. Like other mitochondrial components, the mitochondrial genome is also prone to accumulating damage. However, persistent mtDNA replication stress does not trigger bulk mitochondrial removal but rather leads to the degradation of mitochondrial nucleoids in the endosomal compartment, serving as a selective degradation route for the mitochondrial genome (*13*).

The existence of alternative or cargo-specific mitophagy pathways highlights the complexity and adaptability of cellular systems. Dysfunctional mitochondrial pieces can be precisely eliminated by preserving the rest of the mitochondrial network. Different studies showed that these pathways share components with the vesicle trafficking system. For instance, in an oxidative environment, the retromer induces the formation of MDVs containing oxidized cargo and directed to peroxisomes (*43*). Nevertheless, although the retromer and the mitochondrial genome appeared in cellular degradation compartments upon mtDNA stress (*13*), it was not clear whether this pathway followed MDVs.

To identify the specific mechanism involved in the mitochondrial genome turnover upon mtDNA replications stress, we used directed proximity proteomics between mitochondria and endosomes. Consistent with our previous findings, we detected an enrichment of vesicle and lysosomal-related proteins, including RAB10, ATP6V0D1, MYO1B, BIRC6, and the retromer component VPS29. All these proteins participate in lysosomal maturation and acidification (*34*, *38*, *44*–*46*), suggesting a prominent role of lysosomes in the elimination of the mitochondrial genome.

Our data support a model where the events following mtDNA stress proceed through parallel pathways, strongly regulated by the vesicular system. mtDNA replication stress primarily leads to mitochondrial fragmentation, a process that may aid in the separation of dysfunctional mitochondrial components. Thus, the fission of the mitochondrial network is inhibited in cells expressing the dominant negative allele of RAB10^T23N^ and in retromer-deficient cells. Nevertheless, the role of these two candidates in adapting mitochondrial dynamics upon stress is not new. On the one hand, RAB10, traditionally associated with vesicle transport and endoplasmic reticulum morphology (*47*), also controls mitochondrial turnover upon depolarization (*39*). On the other hand, the retromer induces mitochondrial fragmentation in a DRP1/DLP1-dependent manner. Thus, the dominant negative mutation VPS35^D620N^ causes a VPS26-dependent mitochondrial fragmentation due to an enhanced turnover of mitochondrial DRP1 (*48*). Consistently, VPS35 KO in HeLa cells suppressed the mitochondrial fragmentation caused by mtDNA replication stress. The fragmentation of the network was recovered upon reexpression of VPS35, independently of the accessory subunits VPS29 or VPS26.

**Fig. 9.**
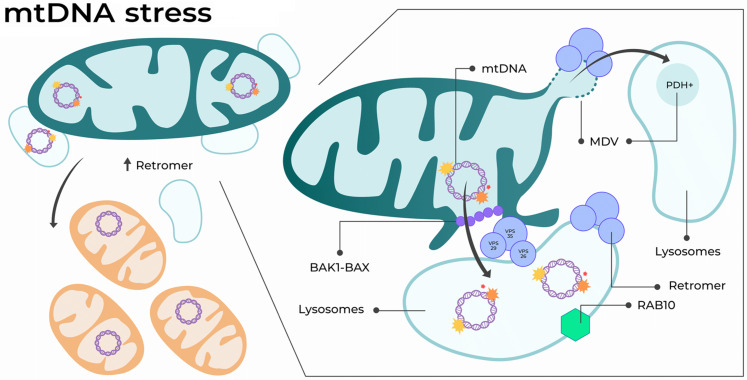
Proposed model for retromer function upon mtDNA stress. The retromer enhances mitochondrial fragmentation and mtDNA turnover. mtDNA ejection occurs in a BAX-dependent manner, targeting RAB10-VPS35-positive lysosomes, and independent of MDVs. Stimulation of these pathways restores mitochondrial function and mitigates defects associated with mtDNA damage in vivo.

Furthermore, mtDNA replication stress triggers the formation of canonical MDVs containing the mitochondrial matrix component PDH. The formation of these vesicles also depends on the retromer and requires the mitochondrial fission protein DRP1 and transport protein Miro1, both critical for the formation of MDVs (*29*, *49*). Notably, these matrix-derived vesicles are distinct from the recently described VDIMs, which form independently of DRP1 (*11*). Unexpectedly, the delivery of the mtDNA to the endosomes does not follow an MDV-related path. In contrast, mtDNA turnover depends on the retromer and, at least, on the Bcl-2–associated X protein BAX. Thus, the recruitment of retromer-vesicles to the mitochondrial surface might activate the formation of BAX-BAK1 macropores, enabling mitochondrial inner membrane herniation and extrusion of big mtDNA fragments (*50*, *51*), followed by vesicle capture. Then, the retromer and other vesicle-related proteins such as RAB10 may promote lysosomal maturation and the degradation of damaged mtDNA (Fig. 9).

Because the overexpression of the retromer core component VPS35 increases mtDNA extraction in vitro, we hypothesized that this would also be beneficial to eliminate mitochondrial burden in vivo. Noteworthy, overexpression of VPS35 has been shown to restore cellular homeostasis associated with lysosomal dysfunction (*52*, *53*). Thus, the presence of ΔmtDNA changes the mitochondrial redox state, which is translated into activation of detoxifying pathways. These changes activate a specific transcriptome profile to counteract cellular damage but also to modify mitochondria structure. Thus, besides the up-regulation of genes for glutathione metabolism and innate immune response, we detected up-regulation of many transcripts encoding for membrane carriers, such as members from aquaporin (*Drip*), ABCB transporters (*Mdr49* and *Mrp4*), ion channels (*aralar1*, *MME1*, *sCaMC*, and *TrpA1*), and mitochondrial proteases (*Lon*, *Afg3L2*, and *Spg7)*. The last three ones are all involved in the degradation of damaged mitochondrial proteins to maintain mitochondrial integrity (*54*). In this context, overexpression of *Vps35* in *Drosophila* restores the mitochondrial defects associated with damaged mtDNA while also restoring the cellular transcriptome, thereby suggesting a recovery of cellular health (Fig. 9).

Together, our data underscore the intricate relationship between mitochondria and lysosomes and how these two organelles coordinate to maintain cellular health. Accordingly, mitochondrial impairment leads to an increase in lysosomal biogenesis, lysosomal acidification, and mitochondrial turnover (*55*). Similarly, we found that, in cells, expression of VPS35 does not influence macroautophagy, but directly impacts on lysosomal acidification upon mtDNA stress burden.

In conclusion, we have provided a mechanistic view of a complex mechanism. mtDNA replication stress follows a sophisticated downstream response that synergizes to prevent the accumulation of damage in the mtDNA and ensure mitochondrial integrity. First, we identified a prominent role of the vesicular system in controlling mitochondrial dynamics. Second, MDVs directed to the lysosomal compartment eliminate the mitochondrial matrix. Last, leaked mtDNA requires the formation of mitochondrial BAX membrane pores. However, the expelled DNA does not exist free in the cytoplasm and is captured in a recycling organelle. We demonstrate in vivo that the retromer can drive the elimination of mutated mtDNA, removing the source of cellular burden and thereby modulating the downstream response. Hence, our data confirm that modulation of VPS35 levels is an effective strategy to counteract mitochondrial damage associated with ΔmtDNA.

## MATERIALS AND METHODS

### Study design

The primary goal of this study was to understand how the mitochondrial genome ends up in recycling organelles upon mtDNA replication stress. We first designed an approach to decipher the proximity proteome of mitochondria and endosomes. To interfere with mtDNA replication, we use the expression of a missense mutation of the mtDNA helicase, knowing that, in replicative cells, it leads to lysosomal-dependent mtDNA depletion (*13*). Our results brought up a subset of candidate proteins involved in the regulation of this process, highlighting the lysosomes. Thus, we selected the retromer and evaluated its role in mitochondrial dynamics and the extraction mechanisms for mitochondrial matrix components. Last, we assayed in vivo the ability of the retromer to increase mitochondrial turnover, for which we used a *Drosophila* model with specific alteration in the mtDNA.

### Plasmid generation

RAB10 plasmids were obtained from Addgene (WT, Addgene #49472; Q68L Addgene #49544; and T23N Addgene #130885). RAB10^T23N^ was subcloned in pEGFP-C1 using Bgl II and Eco RI. VPS35-EGFP and VPS35-CFP were generated by subcloning VPS35 from pLenti/V5/DEST-VPS35 plasmid (Addgene #21691) in pEGFP-N1 and pECFP-N1 plasmid using Xho I and Bam HI sites. Twinkle *Homo sapiens* open reading frame (ORF) was subcloned from Twinkle-APEX2-V5 (Addgene #129705) in pN1-mCherry using Bgl II and Eco RI sites. VPS35 p.D620N and Twinkle p.K319E were generated by site-directed mutagenesis. VPS29-YFP (yellow fluorescent protein) was obtained from Addgene (#174520) and subcloned in pEGFP-C1 using Eco RI and Bam HI cloning sites. MitoKeima was amplified from mKeima-Red-Mito-7 (Addgene #56018) and subcloned in pLenti-CMV-Puro in EcoR V sites. All generated plasmids were verified by Sanger sequencing. To generate stable cell lines, VPS35 or VPS35 p.D620N ORFs were subcloned in pLenti/V5/DEST using EcoR V sites. mtDNA-Kaede plasmid was a gift from T. Shutt (University of Calgary, Canada).

### Generation of HeLa cells KO cell lines

All KO cell lines were generated by CRISPR-Cas9 and cloning of specific guide RNAs (gRNAs) in pSpCas9(BB)-2A-Puro (PX459) V2.0 (Addgene #62988) in the Bbs I site. All gRNAs were designed using http://crispor.tefor.net/crispor.py. *VPS35* KO was generated by targeting exon 13 (ATATGAATTCATGTCC); *VPS26A* was knocked out with a gRNA specific for exon 4 (GCACCGATGTAAGATTCATA) and VPS29, by targeting the isoform 1 (GGCAAACTGTTGCACCGGTG), isoform 2 (GGACATCAAGTTATTCCATG), and isoform 3 simultaneously (GACTATCTCAAGACTCTGGC). Miro1 (*RHOT1*) KO was generated by simultaneously targeting exon 10 (GGGACTGTGCTTCGACGATT) and exon 12 (GTGGCCCCCAAGGTATGTAA). In all cases, transfected cells were selected using puromycin (2.5 μg/ml; Sigma-Aldrich, #P8833) for 72 hours after transfection, followed by isolation of monoclonal cell lines.

### Cell culture and chemical treatments

Cells were maintained in Dulbecco’s modified Eagle’s medium (DMEM) with glucose (4.5 g/liter) + GlutaMAX (Gibco, #10566016) supplemented with 10% fetal bovine serum (FBS), 1x Pen/Strep (Gibco, #15070063), and Plasmocin (2.5 μg/ml; InvivoGen, #ant-mpp). Transfection of HeLa cells was achieved using FuGENE HD (Promega, #E2311) following the manufacturer’s instructions and changing the growing medium 6 hours after transfection. The lysosomal activity was inhibited with 10 μM chloroquine (Sigma-Aldrich, #C6628) for 4 hours where indicated. Oxidative damage was induced by treating cells with 200 μM H_2_O_2_ in a complete medium for 4 hours. For stimulating mitochondrial metabolism, cells were grown overnight in nonglucose DMEM supplemented with 10 mM galactose (Sigma-Aldrich, #1287700), 10% FBS, 1x Pen/Strep, 1x GlutaMAX (Gibco, #35050061), and 1x pyruvate (Gibco, #11360070). For inhibiting mitochondrial membrane pore formation, 24 hours after transfection, cells were treated with 25 μM VBIT-12 (MedChemExpress, #HY-135885) or 100 μM Bax Inhibitor peptide V5 (MedChemExpress, #HY-P0081) alongside with chloroquine for 4 hours prior to fixation.

For the generation of stable cell lines, HeLa cells were transduced with pLenti/V5/DEST-VPS35, pLenti/V5/DEST-VPS35 p.D620N, and pLenti-CMV-Puro-mitoKeima using psPAX2 and pMD2.G as helper plasmids and HEK293 cells for viral packaging. The supernatant containing lentivirus was filtered through a 0.45-μm filter and supplemented with polybrene (10 μg/ml; Sigma-Aldrich, #H9268). Transgenic expression of VPS35 was confirmed by Western blot. Transduced clones were selected and maintained in a medium containing blasticidin S (20 μg/ml; InvivoGen, #ant-bl-05) for VPS35 overexpression cell lines and puromycin (2.5 μg/ml) for mitokeima. HeLa DRP1 KO cells were kindly provided by M. Escobar (University of Cologne, Germany).

### *Drosophila* stocks

The Gal4/UAS system was used for tissue-specific expression (*56*). All *Drosophila* experiments were carried out using *A58-Gal4* driver lines to induce UAS-constructs in the epidermis of *Drosophila* larvae (*57*). *A58-Gal4* is expressed in the epidermis from the first larval instar stage onward (*42*, *58*, *59*). UAS-constructs used in this work are as follows: *UAS-mitoT4lig*, *UAS-mitoAflIII* (BL 84979), UAS-mitoAflIII, UAS-mitoT4lig/CyO (kindly provided by B. A. Hay) (*41*), and *UAS-Vps35.HA* (*60*) (BL67152). All stocks were in a white-eyed genetic background and maintained at 25°C under a 12-hour/12-hour light/dark cycle on standard fly food. All crosses were set up and maintained at 18°C under a 12-hour/12-hour light/dark cycle on standard fly food. The complete genotypes of all strains generated and used in this study are listed in table S1. All molecular experiments using *Drosophila* were conducted using complete larvae, excluding transcriptome analysis, where larval epidermis was isolated.

### mtDNA copy number and qPCR

Total DNA was isolated using the DNeasy Blood & Tissue Kit (Qiagen, #69504) according to the manufacturer’s instructions. Twenty nanograms of total DNA was used for the analysis of threshold amplification differences between mtDNA and nuclear DNA [delta C(t) method]. For human (HS) cell–based analysis, mtDNA copy number was obtained with oligos amplifying the mitochondrial gene *ND1* and the nuclear gene *APP*.

For cytosolic mtDNA detection, HeLa cells stably expressing VPS35 or VPS35^D620N^ were transfected with TWNK^K319E^-mCherry. Forty-eight hours posttransfection, cells were collected and equally divided for internal control and fractionation experiments. The total DNA for internal control samples was obtained by solubilizing cell pellets in 10 mM NaOH, followed by boiling at 98°C for 30 min and neutralization of the pH with Tris (pH 8) to a final concentration of 150 mM. For mitochondria-free fractions, cell pellets were incubated in digitonin (25 μg/ml; Roth, #4005.2), 150 mM NaCl, and 50 mM Hepes (pH 7.4) for 10 min on ice. Unbroken cells and mitochondria were cleared by sequential differential centrifugation first at 1000*g* and resultant supernatant at 14,000*g*. DNA was further isolated using the DNeasy Blood & Tissue Kit and eluted in equal volumes. Three microliters of pure DNA was used to amplify the nuclear gene *APP* and mitochondrial genes *ND1* and *ND5* in both fractions. Cytoplasmic content of mtDNA was obtained after normalization of each Ct value from mitochondria-free fractions to normalized value of total sample Ct (Ct Mitochondrial^MITOC.Free^) − (Ct Mitochondrial^TOTAL^ − Ct Nuclear^TOTAL^) = DDCt. Fold change (FC) values were obtained with 2^−DDCt.

For mtDNA heteroplasmy quantification and mtDNA copy number in *Drosophila*, 20 ng of total DNA isolated from five larvae was used to amplify *ND5* and *CytB*, mitochondrial genes outside and inside the deleted region, respectively, and *3RTub* (TUBB) as a nuclear gene. Both Ct values from mitochondrial genes were normalized with the Ct for *TUBB* and the ratio between *CytB/ND5* was calculated. Quantitative real-time PCR for gene expression was performed using cDNA retrotranscribed from 1 μg of total RNA using PowerUP SYBR green (Thermo Fisher Scientific, #A25780).

All qPCR experiments were performed in Quant Studio 1 (Thermo Fisher Scientific) and PowerUP SYBR Green (Thermo Fisher Scientific, #A25780). Conventional PCR was performed using either cDNA or total DNA, specified in the corresponding figure. Primer sequences are specified in table S2.

### Western blot and immunofluorescence

For protein isolation, cells were pelleted at the indicated time points and resuspended in radioimmunoprecipitation assay (RIPA) buffer [150 mM NaCl, 1% Triton X-100, 0.1% SDS, 50 mM tris-HCl (pH 8), 1 mM EDTA, and 0.5% Na-deoxycholate] containing protease inhibitor (Sigma-Aldrich, #11697498001). Protein concentration was quantified using Bradford reagent (Bio-Rad, #5000001), and equal amounts of protein were loaded onto SDS–polyacrylamide gel electrophoresis (PAGE), transferred to polyvinylidene difluoride membrane (Bio-Rad), blocked in TBST–5% fat-free milk, and blotted with the indicated antibodies. For Western blot involving *Drosophila*, five L3 larvae were homogenized in lysis buffer [150 mM NaCl, 1% Triton X-100, 50 mM tris-HCl (pH 8), 0.01% NP-40, and 1 mM EDTA] containing a protease inhibitor, using a Teflon pestle. Equal amounts of proteins were directly loaded onto SDS-PAGE and proceeded as described above.

Antibodies used for Western blot were as follows: polyclonal α-SDHB (10620-1-AP; 1:1000), α-TOM20 (11802-1-AP; 1:1000), α-optineurin (10837-1-AP; 1:1000) α-LC3B (14600-1-AP; 1:1000), α-GFP (50430-2-AP; 1:1000), α-VPS26A (12804-1-AP; 1:1000), α-Miro1 (#84055-5-RR; 1:1000), and monoclonal α-GAPDH (60004-1-Ig; 1:4000), α-GFP (66002-1-Ig; 1:1000), and α-p62 (66184-1-Ig; 1:1000) from Proteintech; polyclonal α-RAB5 (ab109534; 1:1000), α-VPS29 (ab236796; 1:1000), and monoclonal α-ATP5A (ab14748; 1:1000) from Abcam; polyclonal α-LAMP1 (#9091; 1:1000), α-V5 (#13202; 1:1000), and monoclonal α-V5 (#80076; 1:1000) from Cell Signaling; monoclonal α-VPS35 (sc-374372; 1:1000) and α-RAB5 (sc-46692; 1:500) from Santa Cruz; monoclonal α-DLP1(DRP1) from BD Bioscience (#611112; 1:500). All membranes were incubated with appropriate secondary antibodies combined with horseradish peroxidase (HRP) (Jackson ImmunoResearch; goat anti-mouse #115-035-003; goat anti-rabbit #111-035-003), developed using Western Lighting Plus ECL (PerkinElmer, #NEL104001EA), and visualized using LAS500 CCD camera (GE Healthcare).

For immunofluorescence, cells were seeded on glass coverslips, fixed for 15 min in 4% paraformaldehyde/phosphate-buffered saline (PBS), and stored at 4°C until use. Permeabilization was achieved using 0.2% Triton/PBS followed by 1-hour blocking (5% fat-free milk, 10% FBS, 0.1% Triton, and 0.5% BSA in PBS). Primary antibodies were incubated overnight at 4°C. Antibodies used for immunofluorescence were as follows: polyclonal α-TOM20 (11802-1-AP; 1:1000) and α-VPS26A (12804-1-AP; 1:1000) from Proteintech; monoclonal α-dsDNA (ab27156; 1:1000), α-PDHX (ab110333; 1:500), α-ATP5A (ab14748; 1:500), and polyclonal α-RAB5 (1:500) from Abcam; polyclonal α-LAMP1 (#9091; 1:500), α-V5 (#13202; 1:500), and monoclonal α-V5 (#80076; 1:500) from Cell Signaling; monoclonal α-VPS35 (sc-374372; 1:500) from Santa Cruz. After washing the coverslips with PBS, samples were incubated with appropriate secondary antibodies (1:1000, 1 hour at room temperature) from Thermo Fisher Scientific: goat α-rabbit combined with Alexa-405 (#A-31556), goat α-rabbit Alexa-488 (#A-11008), goat α-mouse Alexa-488 (#A-11001), goat α-rabbit Alexa-546 (#A-11035), goat α-mouse Alexa-546 (#A-11030), goat α-rabbit Alexa-647 (#A-21245), and goat α-mouse Alexa-647 (#A-21235) Coverslips were mounted using Fluoromount containing 4′,6-diamidino-2-phenylindole (DAPI) (Thermo Fisher Scientific, #00-4959-52).

### Confocal microscopy and flow cytometry

For confocal microscopy, images were acquired using a Leica SP8 microscope with a 63x/1.40 oil PL Apo objective and a PerkinElmer Spinning-disk confocal microscope UltraVIEW VoX with 60x objective. All images were deconvoluted using ImageJ (NIH) before analysis to increase resolution. Brightness was adjusted equally in the entire image. All image analyses were performed using ImageJ (NIH). Quantification of mitochondrial morphology was achieved using as particle analysis tool with nonexclusion criteria. Mitochondrial vesicle quantification was performed using an internal IMAGE J plugin by counting TOM20−/PDH+ or TOM20−/dsDNA^+^ particles. Manders’ correlation coefficient was calculated using the JACOP plugin (*61*). The fluorescence profile was obtained by drawing a line covering 50 pixels and using the RGB Profiler plugin.

For LysoTracker experiments, cells were seeded onto μ-Dish 35 mm, high Glass Bottom (Ibidi, #81158) and transfected with TWNK^K319E^-mCherry plasmid. Twenty-four hours after transfection, cells were loaded with 50 nM LysoTracker Green (Thermo Fisher Scientific, #L7526) for 30 min, following a medium change and immediately imaged using the spinning disk at 37°C. Only cells transfected with TWNK^K319E^-mCherry were considered for the analysis.

Analysis of bulk mitophagy was performed in cells stably expressing the mitophagy reporter Mitokeima. Flow cytometry analysis was performed on a CytoFLEX-S (V4-B2-YG4-R3), Beckman-Coulter (Acquisition Software CytExpert V2.4.0.28). After setting the cell gate (FSC-A/SSC-A), FSC-H and FSH-W were used for doublet discrimination. The signals from the mt-Keima construct were measured in the Violet 610 (excitation laser 405 nm; 610/20 bandpass filter) and the ECD (excitation laser 561; 610/20 bandpass filter) detector channels.

### Electron microscopy

The epidermis from *Drosophila* L3 larvae was isolated and immersion fixed in 2% formaldehyde, 2% glutaraldehyde, and 3 mM CaCl_2_ in 0.1 M sodium cacodylate buffer for 48 hours. Samples were washed with 0.1 M sodium cacodylate buffer and postfixed with 2% OsO_4_ in 0.1 M cacodylate buffer, washed 3x5 min with ddH_2_O, followed by dehydration in an ascending ethanol series for 15 min each (50, 70, 90, and 3x100%) at 4°C. Samples were incubated for 15 min with 50% ethanol/propylene oxide, followed by two times 15 min pure propylene oxide. Then, samples were infiltrated with a mixture of 50% Epon/propylene oxide and 75% Epon/propylene oxide for 2 hours each and pure Epon overnight at 4°C. The next day, samples were incubated with fresh Epon for 2 hours and placed into flat embedding molds and cured at 60°C.

Ultrathin sections of 70 nm were cut using an ultramicrotome (Leica Microsystems, UC6) and a diamond knife (Diatome, Biel, Switzerland) and stained with 1.5% uranyl acetate and 3% Reynolds lead citrate solution. Images were acquired using a JEM-2100 Plus Transmission Electron Microscope (JEOL) operating at 80 kV equipped with a OneView 4K camera (Gatan).

For CLEM, cells were seeded onto carbon-coated plates containing a numerated grid. Cells were transfected with TWNK^K319E^-mCherry and VPS35-CFP plasmids as described previously. The lysosomal function was blocked for 4 hours with 10 μM chloroquine. One hour before fixation, cells were loaded with 1x SYBRGold (Thermo Fisher Scientific, #S11494) and 250 nM PK mito Deep Red (Spirochrome, #SC055). Cells were fixed for 30 min at room temperature and 30 min at 4°C in 2% glutaraldehyde, 2.5% sucrose, and 100 mM CaCl_2_ in 0.1 M Hepes (pH 7.4) and washed with 0.1 M Hepes buffer. Transfected cells showing cytoplasmic DNA were scanned using the SP8 confocal microscope (Leica) with 63x/1.40 oil objective, and bright-field images were used to localize the coordinates of cells of interest. Following light imaging, cells were incubated with 1% Osmiumtetroxid for 30 min at 4°C, washed in 0.1 M cacodylate buffer, and dehydrated using ascending ethanol series (50, 70, 90, and 100%) at 4°C. Cells were infiltrated with a mixture of 50% Epon/ethanol for 1 hour, 66% Epon/ethanol for 2 hours, and pure Epon overnight at 4°C. TAAB capsules filled with Epon were placed upside down onto the glass bottom and cured for 48 hours at 60°C. Glass bottom was removed by alternatingly putting the dish into boiling water and liquid nitrogen. Block face was trimmed to the previously noted coordinates using a 90° trim tool (Diatome, Biel, Switzerland), and ultrathin sections of 70 nm were cut using an ultramicrotome (Leica Microsystems, UC6) and a diamond knife (Diatome, Biel, Switzerland) and stained with 1.5% uranyl acetate for 15 min at 37°C and lead citrate solution for 4 min.

For electron tomography, ultrathin sections of 300 nm were cut and incubated with 10 nm of protein A gold (CMC, Utrecht) diluted 1:60 in ddH_2_O. Sections were stained with 2% uranyl acetate for 20 min and Reynolds lead citrate solution for 3 min. Images for the tilt series were acquired using Serial EM with a pixel size of 1.108 nm from −60° to 60° with a 1° increment on the same JEM-2100 Plus Transmission Electron Microscope (JEOL) operating at 200 kV. Reconstruction was done using Imod, MiB, and Imaris.

### Proximity biotinylation

Proximity biotinylation was performed in transduced cells expressing Split TurboID constructs RAB5C-SplitNt-V5 and SAMM50-SplitCt-HA. Cells expressing only SAMM50-SplitCt-HA were used as a negative control.

For protein isolation, HEK293 cells were incubated for 4 hours with 250 μM biotin. For inducing mtDNA damage, cells were previously transfected with TWNK^K319E^-mCherry 24 hours before the biotin incubation. Pellets were scraped from plates right after the biotin incubation and solubilized in RIPA buffer. Three hundred micrograms of total protein extracts were purified using Streptavidin Sepharose High-Performance beads (GE Healthcare Bio-Sciences AB). For Western blot analysis, beads were washed three times with RIPA buffer, resuspended in 2x Laemmli buffer, boiled at 95°C for 5 min, and loaded onto SDS-PAGE. Biotinylated proteins were visualized with α-Streptavidin–HRP antibody (Jackson ImmunoResearch, 1:2000, #016-030-084). For mass spectrometry, beads were washed three times with ABC buffer [50 mM ammonium bicarbonate (pH 7.8)] and incubated for 10 min with 50 μl of urea buffer (8 M urea), 1 hour with 5 mM dithiothreitol, and 30 min with 40 mM indole-3-acetic acid at room temperature and in the dark. Protein digestion was achieved by incubating with Lys-C in a ratio of 1:100, for 2 to 3 hours. Samples were diluted with ABC buffer to a final urea concentration of 2 M and incubated with trypsin overnight in a ratio of 1:100. Digestion was stopped with 1% formic acid and peptides loaded into preequilibrated stage tips and stored at 4°C until used for mass spectrometry analysis.

### Liquid chromatography–mass spectrometry analysis

Affinity-enriched proteomics samples were analyzed in positive mode using data-dependent acquisition either by an Easy-nLC 1000–Q Exactive Plus or an Easy-nLC 1200–Orbitrap Eclipse tribrid system (Thermo Fisher Scientific). Online chromatography was directly coupled to the mass spectrometry systems using a nanoelectrospray ionization source. Peptides were separated by reversed-phase chromatography with a binary buffer system of buffer A (0.1% formic acid in water) and buffer B (0.1% formic acid in 80% acetonitrile) using a 60-min chromatographic gradient. Separation was performed on a 50-cm-long in-house packed analytical column filled with 1.9 μM C18-AQ Reprosil Pur beads (Dr. Maisch). Using the 60-min chromatographic gradient, peptide separation based on their hydrophobicity was performed by linearly increasing the amount of buffer B from initial 13 to 48% over 35 min followed by an increase in B to 95% for 10 min. The column was washed for 5 min, and initial column conditions were achieved by equilibrating the column for 10 min at 7% B. Full mass spectrometry spectra [300 to 1750 mass/charge ratio (*m/z*)] were acquired with a resolution of 70,000, a maximum injection time of 20 ms, and an automatic gain control (AGC) target of 3 × 10^6^. The top 10 most abundant peptide ions were isolated (1.8 *m/z* isolation windows) for subsequent HCD fragmentation (NCE = 28), and tandem mass spectrometry (MS/MS) recorded at a resolution of 35,000, a maximum injection time of 120 ms, and an AGC target of 5 × 10^5^. Peptide ions selected for fragmentation were dynamically excluded for 20 s.

### Data processing and analysis for proteomics

All recorded RAW files were processed with the MaxQuant software suite 75 (1.5.3.8 for IP data, 1.6.14 for pSILAC). For peptide identification and scoring, MS/MS spectra were matched against the mouse UniProt database (downloaded 15 August 2019) using the Andromeda search algorithm 76. For the affinity-enriched samples, multiplicity was set to one and trypsin/P was selected as digestive enzyme. Carbamidomethylation was set as a fixed modification, and methionine oxidation or N-terminal acetylation was selected as a variable modification. Peptides were identified with a minimum amino acid length of seven and a false discovery rate (FDR) cutoff of 1% on the peptide level. Proteins were identified with FDR < 1% using unique and razor peptides for quantification. Label-free quantification (LFQ) was performed using the standard settings of the maxLFQ algorithm. Match between runs was activated.

Statistical analysis and visualization were done with the Perseus (1.6.5) and InstantClue software suites 77 and 78. LFQ intensities of immunoprecipitation samples were log_2_ transformed and filtered for proteins identified in at least two replicates of one condition. Missing values were imputed with the Perseus plugin ImputeLCMD using a deterministic minimal value approach (MinDet, *q* value = 0.001) to simulate the lower detection limits of the mass spectrometer. To evaluate the principal components responsible for the variances between samples, we performed a principal components analysis. Furthermore, we performed a two-sided Student’s *t* test to identify significantly regulated proteins (S0 = 0.1, permutation-based FDR = 0.05, 500 randomizations). If not otherwise indicated, significantly enriched proteins were determined by a combination of FDR-corrected *P* values and log_2_ protein FCs (*q* value < 0.05 and absolute log_2_ FC > 1). Pathway analysis was performed using Metascape (*62*).

### Transcriptomic analysis

For RNA next-generation sequencing, L3 larval epidermis from five larvae was first isolated and pooled. Total RNA was extracted as explained previously, and 2 μg of RNA was sequenced using Poly A + selection. A total of 25 million to30 million reads per sample were obtained using paired-end 100-bp read length (Illumina NovaSeq 6000). A quality control analysis of the RNA-seq raw data was performed with FastQC v0.12.0 (Babraham Bioinformatics) and MultiQC v1.18 software (*63*). 3′ poly(A) tail and the 3′ adapter from Illumina were trimmed with the TrimGalore 0.06.10 tool (Babraham Bioinformatics) to reach a PhredScore greater than 35 and adapter content lower than 1%. Trimmed reads were then mapped onto the *Drosophila melanogaster* genome (Drosophila BDGPG.32, ENSEMBL) by using the RNA-seq aligner STAR 2.7.10b (*64*), followed by a quality assessment of the alignment with Qualimap v2.2.1 (*65*). To estimate gene expression levels, mapped reads for all transcript variants of a gene (gene counts data) were counted with RSEM v1.3.3 software.

Quality control, trimming, mapping, and counting steps were performed in an Ubuntu 22.04.2 LTS environment. Further analysis was run through the RStudio 2023.03.0 +386 (R version 4.2.2) software. Thus, gene counts were annotated with the AnnotationHub Bioconductor package, and four differential expression analyses were performed both by applying the negative binomial distribution with EdgeR-LRT (likelihood-ratio test), EdgeR-QL (quasi-likelihood) (*66*), and DESeq2 (*67*) bioconductor packages or by applying empirical Bayes model using Limma-Voom analysis (*68*, *69*). Genes with a common differential expression among the four methods (cpm < 0.5; *P* < 0.05) were selected for the GSEA with GO pathways by using the clusterProfiler Bioconductor package (*70*) and DAVID Knowledgebase (NIH).

### Statistical analysis

Statistical analysis was conducted using GraphPad Prism version 10 (GraphPad Software Inc., San Diego, CA, USA). The significance of differences among the two or more samples was calculated using unpaired and parametric Student’s *t* test or one-way analysis of variance (ANOVA) test, according to the experimental design and data distribution. The precise number of samples analyzed in each experiment and the respective statistical test used for analysis are reported in figure captions. Statistical significance is determined by *P* values, which are shown in each figure.

## Supplementary Materials

### The PDF file includes:

Figs. S1 to S8 Tables S1 and S2 Legends for data S1 to S3 Legends for movies S1 to S3 Uncropped WB and agarose gels
