# Supplementary material for: Retromer promotes the lysosomal turnover of mtDNA
Source: Sci Adv. 2025 Apr 4;11(14):eadr6415. doi: 10.1126/sciadv.adr6415 (PMC11970507; doi:10.1126/sciadv.adr6415)
Supplement: Supplementary file 1 — Figs. S1 to S8 Tables S1 and S2 Legends for data S1 to S3 Legends for movies S1 to S3 Uncropped WB and agarose gels [file sciadv.adr6415_sm.pdf]

Science Advances
AAAS

Supplementary Materials for

Retromer promotes the lysosomal turnover of mtDNA

Parisa Kakanj *et al.*

Corresponding author: David Pla-Martín, plamartin@hhu.de

Sci. Adv. **11**, eadr6415 (2025)
DOI: 10.1126/sciadv.adr6415

The PDF file includes:

Figs. S1 to S8
Tables S1 and S2
Legends for movies S1 to S3
Legends for data S1 to S3
Uncropped WB and Agarose Gels

Other Supplementary Material for this manuscript includes the following:

Movies S1 to S3
Data S1 to S3

Figure S1

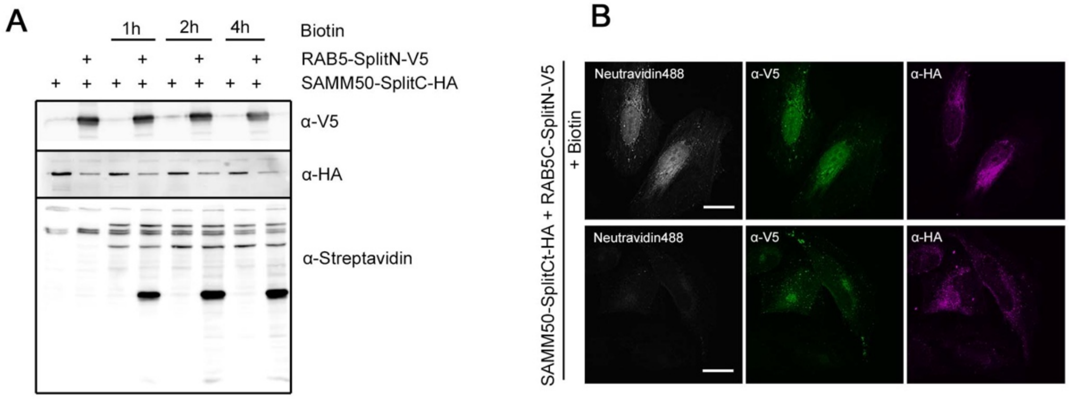

Figure S1. (**A, B)** Reconstitution of biotinylation activity of TurboID by co-expression of RAB5C-SplitN-V5 and SAMM50-SplitC-HA. Biotinylated proteins were detected in (A) western blot with α-Streptavidin-HRP and (B) by immunostaining with Neutravidin 488. α-HA and α-V5 were used to detect SAMM50 and RAB5C respectively. Scale bar, 10 μm

Figure S2

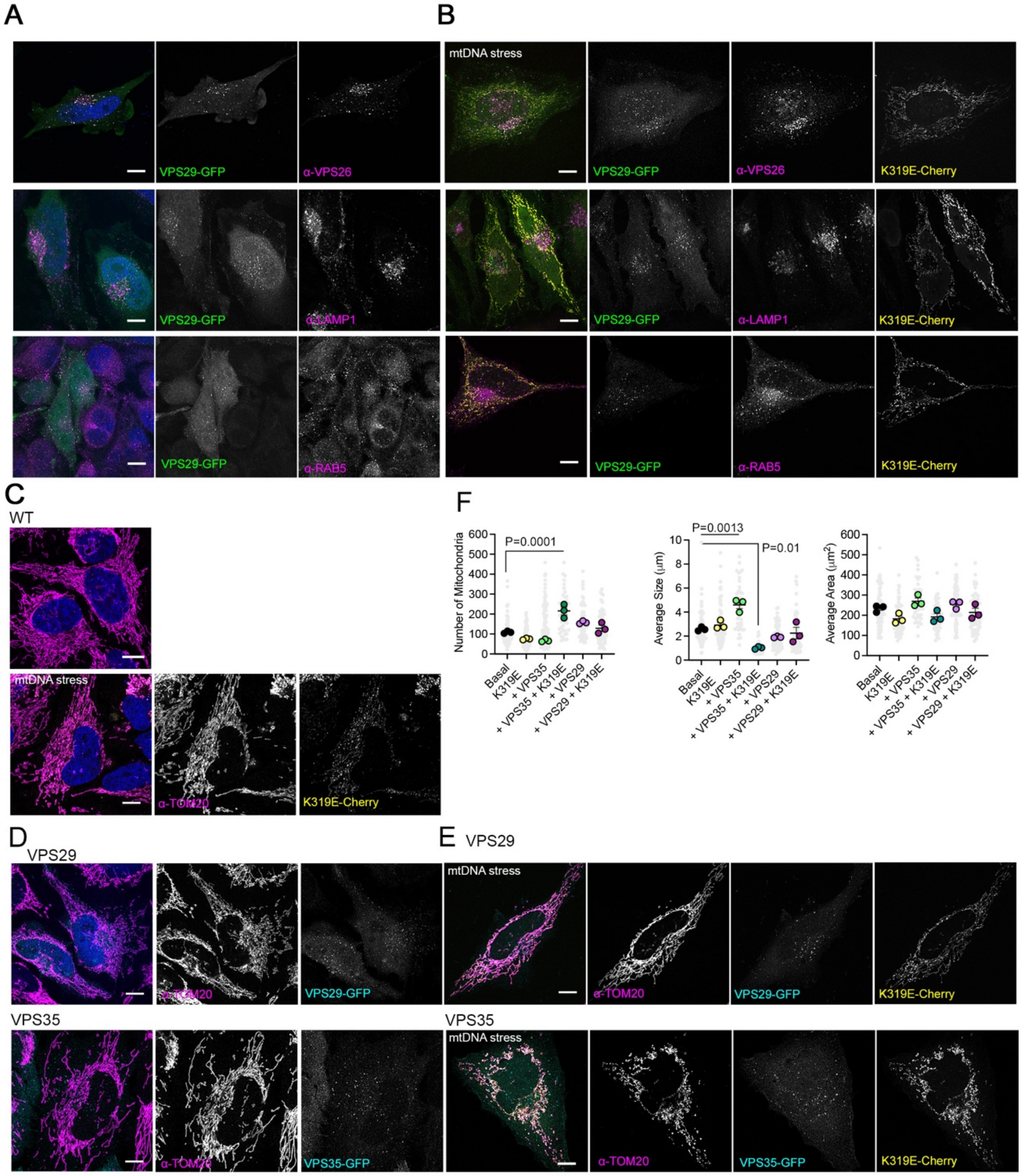

Figure S2. (A, B) Confocal images of HeLa cells expressing VPS29-GFP and (B) TWNK^{K319E}-Cherry labeled with α-VPS26 for the retromer component, α-LAMP1 and α-RAB5 for late endosomes and early endosomes respectively. (**C-E**) Mitochondrial morphology in wt HeLa cells and cells expressing TWNK^{K319E}-Cherry, VPS29-GFP and VPS35-GFP, labelled with α-TOM20. (**F**) Quantification of the mitochondrial morphology.

n=3, >20 cells replicate. P values were calculated using One-Way ANOVA with Tukey correction for multiple comparisons. Scale bar, 10 μm. Data is presented as mean ± SEM.

Figure S3

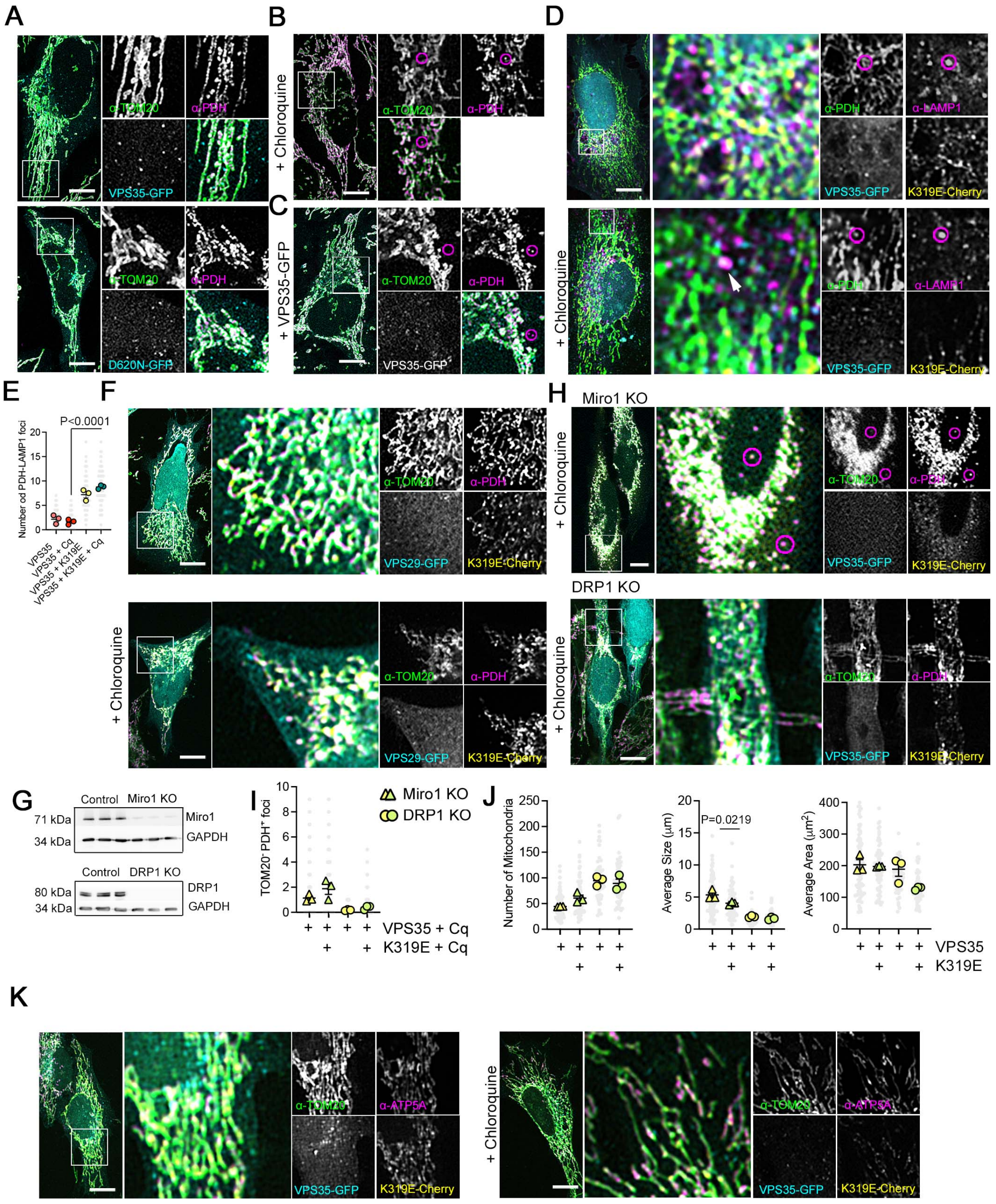

Figure S3. (A-C) Confocal images of cells labelled with α-TOM20 and α-PDH for the analysis of TOM20- PDH+ foci in the steady state. TOM20- PDH+ foci are encircled in magenta. (**D, E**) Analysis of colocalization foci between α-PDH and α-LAMP1 in cells expressing VPS35-GFP and TWNK^{K319E}-Cherry. LAMP1+ PDH+ foci are encircled in

magenta. (**F**) Cells expressing VPS29-GFP and labeled with α-TOM20 and α-PDH. (**G**) Western blot analysis of Miro1 and DRP1 KO in HeLa cells. α-GAPDH was used as a loading control. (**H, I**) Analysis and quantification of TOM20- PDH+ vesicles in Miro1 and DRP1 KO HeLa cells. n=3, > 20 cells per replicate. (**J**) Quantification of the mitochondrial morphology in Miro 1 and DRP1 KO HeLa cells expressing TWNK^{K319E}-Cherry. n=3, > 20 cells per replicate. (**K**) Immunostaining of cells expressing VPS35-GFP and TWNK^{K319E}-Cherry and labeled with α-TOM20 and α-ATP5A for the presence of vesicles derived from the inner membrane. Where indicated, cells were treated for 4 hours prior to fixation with 10μM Chloroquine. P values were calculated using One-Way ANOVA with Tukey correction for multiple comparisons. Scale bar, 10 μm. Data is presented as mean ± SEM

Figure S4

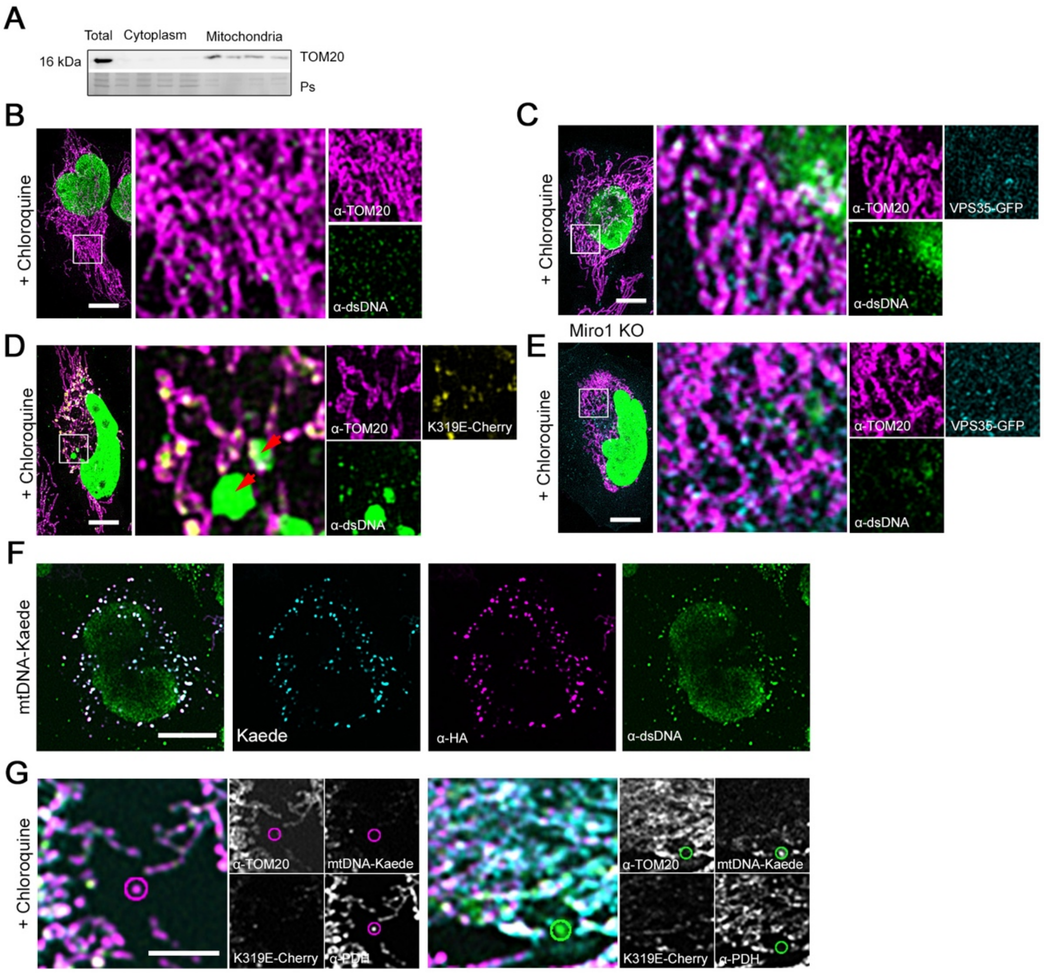

Figure S4. (**A**) Western blot analysis of mitochondria-free fractions used for cytosolic mtDNA qPCR quantification in Figure 4C. Ponceau S was used as a loading control. (**B-E**) Confocal images showing cytosolic dsDNA in (B) wt HeLa, (C) cells expressing VPS35-GFP, (D) TWNK^{K319E}-Cherry and (E) Miro1 KO cells expressing both VPS35-GFP and TWNK^{K319E}-Cherry. Arrows indicate cytosolic dsDNA. (**F**) Cells expressing mtDNA-Kaede plasmid and labeled with α-HA (mtDNA-Kaede tag) and α-dsDNA. TOM20+ PDH-

vesicles are encircled in magenta, while TOM20- mtDNA+ are encircled in green. Where indicated, cells were treated for 4 hours with 10μM Chloroquine before fixation. Scale bar, 10 μm except for (G), 5 μm.

Figure S5

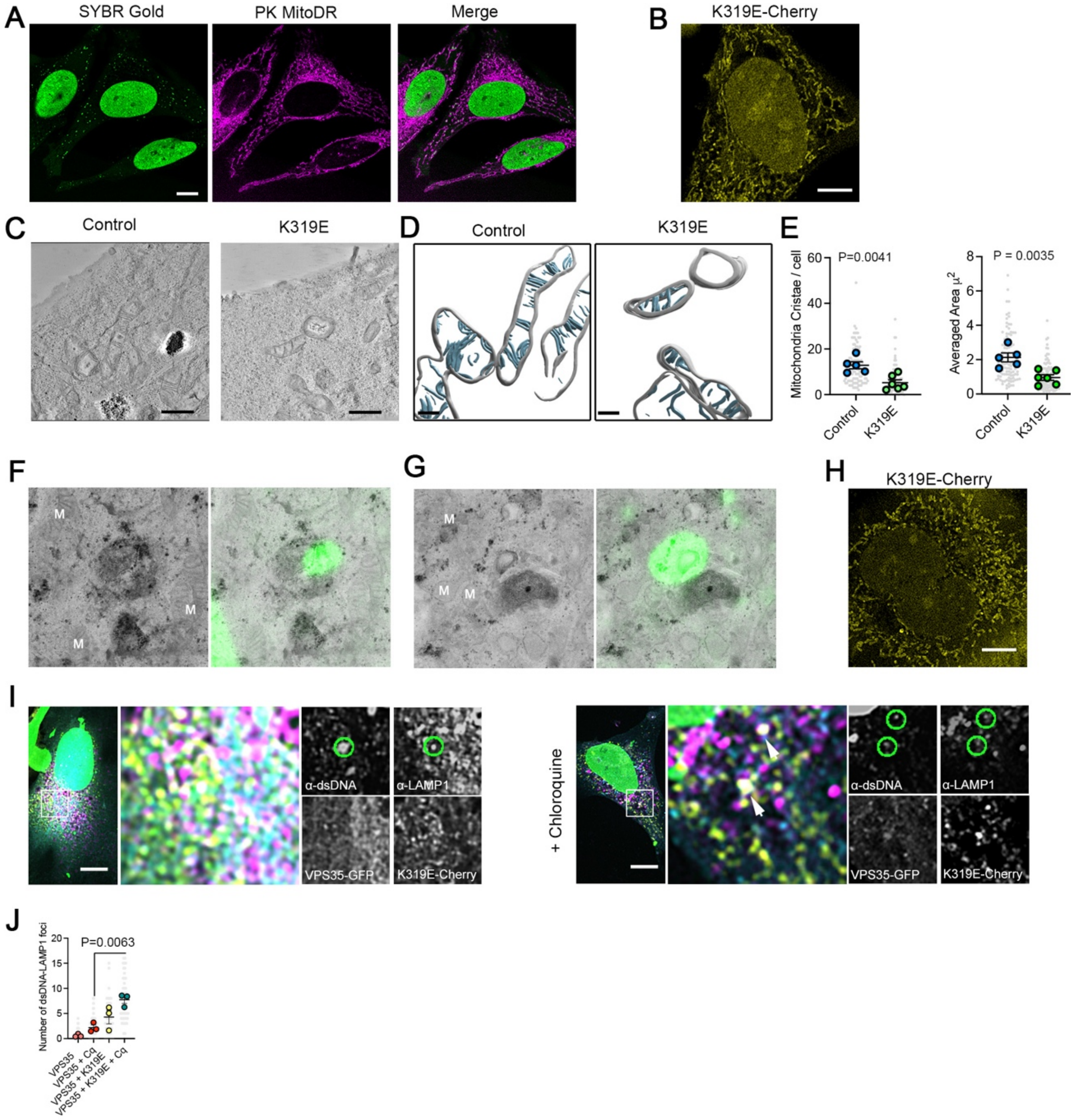

Figure S5. (**A**) HeLa cells loaded with SYBR Gold and PK MitoDeep Red. (**B**) TWNK^{K319E}-Cherry image corresponding to the cell used for CLEM in Figure 5A. (**C, D**) Volumetric reconstitution of electron tomographies from control and TWNK^{K319E}-Cherry cells. (**E**) Quantification of the morphological parameters of mitochondria cristae based on electron microscopy images. (Control, n=5; TWNK^{K319E}, n=6. >10 mitochondria per cell). (**F**) CLEM of cytosolic DNA presented in Figure 5B and C. M, mitochondria. (**H**) TWNK^{K319E}-Cherry

for the cell used for CLEM in Figure 5D. (**I**) HeLa cells expressing VPS35-GFP and TWNK^{K319E}-Cherry and labeled with α-dsDNA and α-LAMP1. Colocalization foci are encircled in green. (**J**) Quantification of colocalization between cytosolic dsDNA and LAMP1. n=3, > 20 cells per replicate. Where indicated, cells were treated for 4 hours before fixation with 10μM Chloroquine. *P* values were calculated using One-Way ANOVA with Tukey correction for multiple comparisons (J) or Student´s T-test (E). Scale bar, 10 μm (A, B, H, I), 1 μm (C), 500nm (D). Data is presented as mean ± SEM.

Figure S6

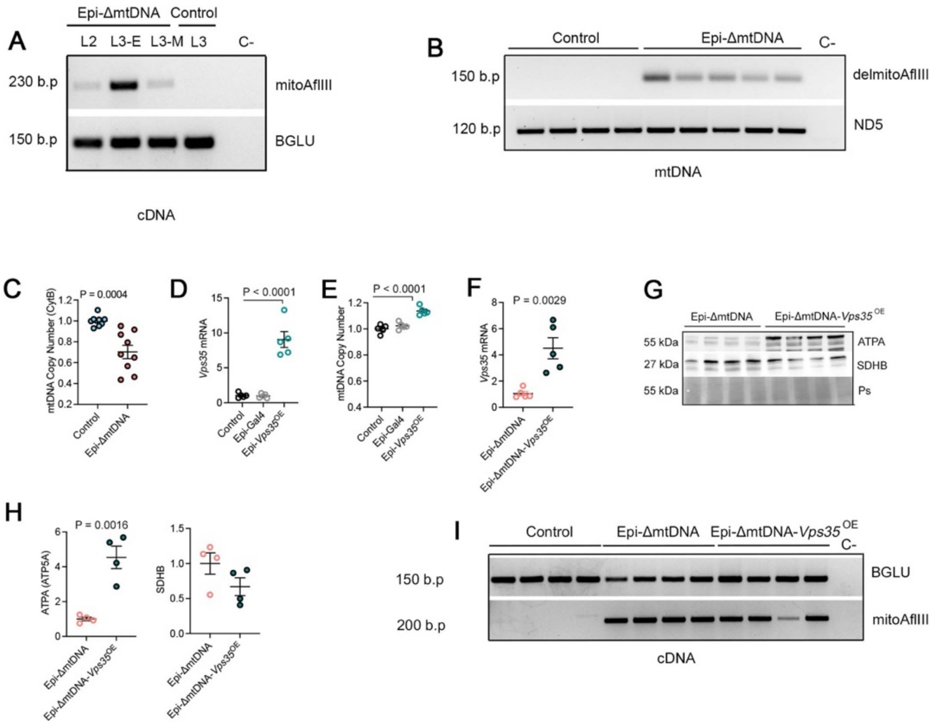

Figure S6. (**A**) Conventional PCR amplification of mtAflIII restriction enzyme in cDNA obtained from RNA isolates of L2 and L3 larvae (E, early; M, mid-stage). mRNA for *BGLU* was used as a housekeeping gene. (**B**) PCR amplification of the mtDNA deletion generated by co-expression of mitoAflIII and mitoT4-Ligase. Primers flank a region separated approximately by 2500 b.p. Upon restriction of the mtDNA and ligation, the region is reduced to approx. 150 b.p. The mtDNA gene *ND5,* located outside the restricted region, was used as a control for mtDNA amplification. (**C**) mtDNA copy number analysis in Epi-ΔmtDNA larvae using *CytB* gene as a reference. (n=9). (**D, E, F**) mRNA quantification and mtDNA copy number analysis in Epi-*Vps35*^{OE}. (n=5). (**G, H**) Western blot and quantification from total protein extracts for ATPA and SDHB for Epi-*Vps35*^{OE}. Ponceau S (Ps) was used as a loading control. (n=4). (**I**) PCR amplification of mitAflIII mRNA in Epi-ΔmtDNA and Epi-ΔmtDNA-*Vps35*^{OE} larvae. mRNA for *BGLU* was used as a housekeeping gene. *P* values were calculated using One-way ANOVA with Tukey correction for multiple comparisons (D, E) and Student´s T-test (C, F, H). Data is presented as mean ± SEM

Figure S7

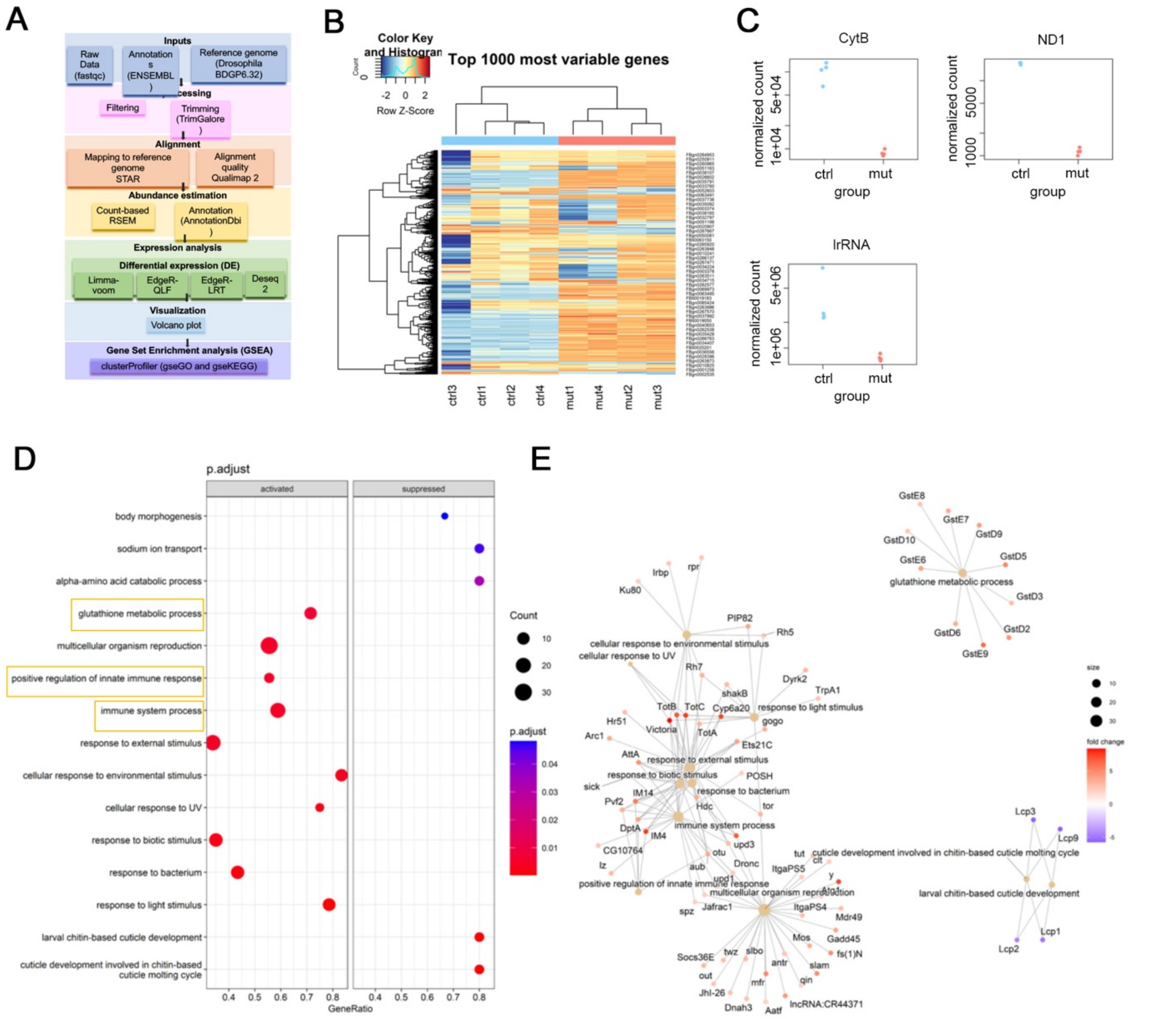

Figure S7. (**A**) Next Generation Sequencing (NGS) analysis flow chart for sequential steps and bioinformatic methods for the raw gene expression quantification in Ubuntu, following by the differential gene expression quantification and the final enrichment analysis using Rstudio software. (**B**) Heatmap of log-CPM values for top 1000 variable genes in each sample. Expression across each gene (row) for all samples (column) ranges from red for high expression to blue for low expression. (**C**) Dotplot evidencing the lower expression of mitochondrial genes *CytB*, *ND1* and *lrRNA* in ΔmutDNA samples. (**D**) Dotplot ranking of the 10 most activated and suppressed GO biological process (BP) sorted by P value < 0.05 between ctrl and ΔmtDNA, generated by gseGO and dotplot. (**E**) Cnetplot for gene association network between the 12 most differentially expressed GO biological process (GO) sorted by p value > 0.05. Orange remarks highlight the biological process of interest for this study.

Figure S8

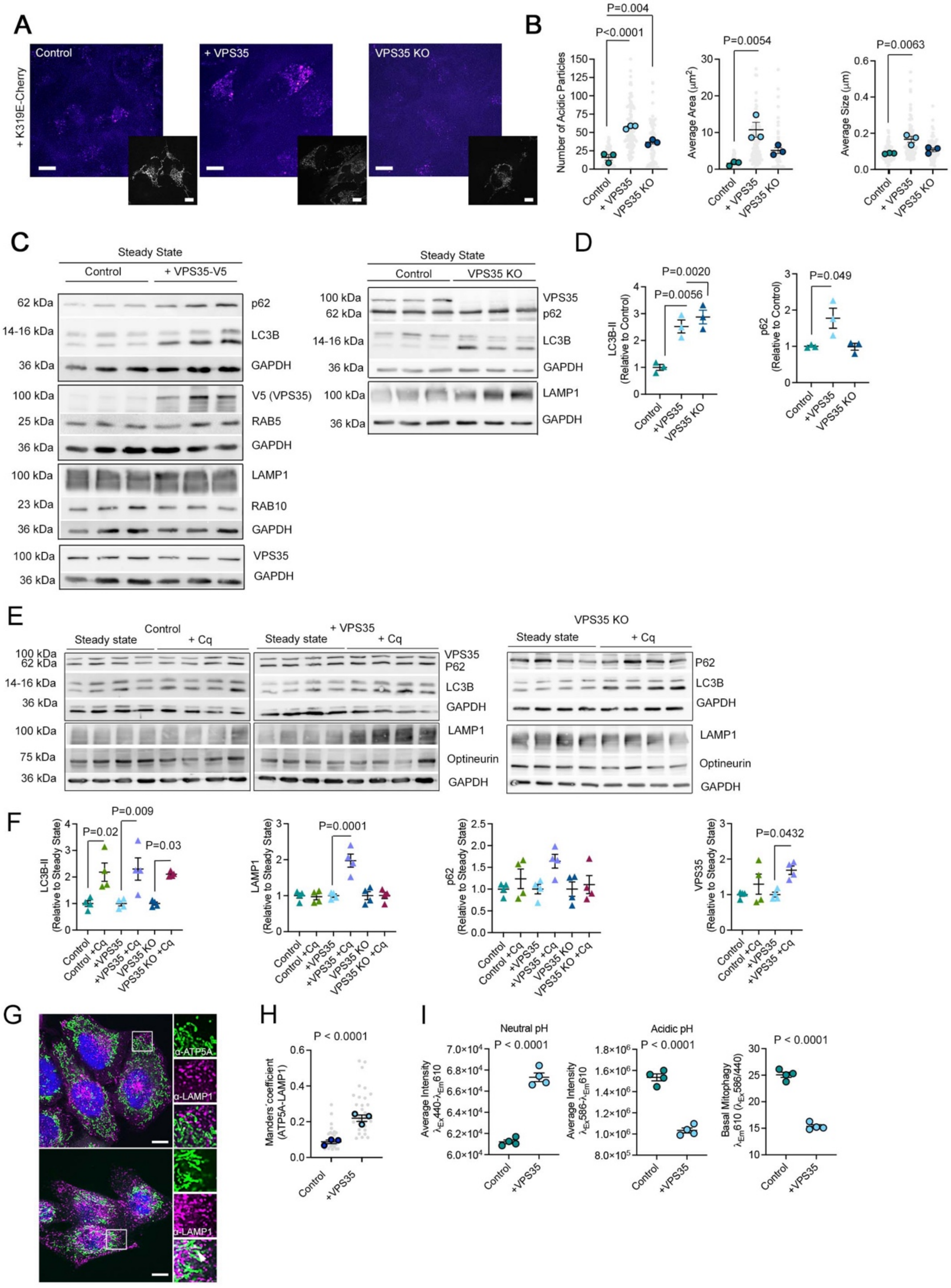

Figure S7. (**A**) Lysotracker staining in VPS35 expressing or VPS35 KO cells, further expressing TWNK^{K319E}-Cherry (insets). (**B**) Morphological quantification of the acidic particles stained by Lysotracker. The analysis was restricted to TWNK^{K319E}-Cherry expressing cells. n=3, > 20 cells per replicate. (**C, D**) Western blot analysis and (D)

quantification for autophagy markers in cells expressing VPS35-V5 and VPS35 KO in the steady state. α-GAPDH was used as a loading control. (**E, F**) Analysis of Autophagy flux and (F) quantification in cells treated with 10 μM Chloroquine (Cq) for 4h. (**G**) α-ATP5A and α-LAMP1 immunostaining in VPS35-V5 cells. Arrows depict colocalization foci. (**H**) Manders´ correlation coefficient between ATP5A and LAMP1 (n=3, 10 images per replicate) (**I**) Quantification of basal mitophagy using mitokeima reporter. After acidification of mitochondria upon lysosomal engulfment, the excitation peak of MitoKeima shifts from 440nm to 586nm, while the fluorescence emission remains stable at 610nm. The average intensity of the fluorescence detected at 610nm when excited at 440 and 586 nm was used to calculate a ratio. P values were calculated using the Student´s T-test (H, I) and One-way ANOVA with Tukey correction for multiple comparisons (B, D, F). Scale bar, 10 μm (A, G). Data is presented as mean ± SEM.

Table S1.

List of genotypes and abbreviations of *Drosophila* lines generated in this study

Genotype abbreviation	Fly genotype
Control	w^{1118}*; +; A58-Gal4 /+*
UAS-Control	w^{1118}*; +;* UAS-Vps35.HA/+<br>*or*<br>w^{1118}*; UAS-mitoT4lig*, *UAS-mitoAflIII/+; +*<br>or<br>w^{1118}*; +; UAS-mitoT4lig*, *UAS-mitoAflIII/+*
Epi-ΔmtDNA	w^{1118}*; UAS-mitoT4lig*, *UAS-mitoAflIII/+; A58-Gal4/+*<br>*or*<br>w^{1118}*; +; A58-Gal4/UAS-mitoT4lig*, *UAS-mitoAflIII*
Epi-*Vps35*^{OE}	w^{1118}*; +; A58-Gal4/* UAS-Vps35.HA
Epi-ΔmtDNA-*Vps35*^{OE}	w^{1118}*; UAS-mitoT4lig*, *UAS-mitoAflIII/+; A58-Gal4/*UAS-Vps35.HA

Table S2.

Primer sequence for *Drosophila*-related samples.

ND5 Dm	gaagtaaagctacatccccaattcg	ggtgagatggtttaggacttgtttc
CYTC Dm	Catatttgtcgagacgttaattatggttg (AflIII site 5´)	atatgaaccgtaataaattcctcgtcc
lrRNA Dm	N/A	Gaattcggcaaaaataatattcgcctg (AflIII site 3´)

3R:TUB Dm	tataagtaaaggcagcagggagac	atctgggtactcttcctctccatc
Vps35 Dm	aaacttttgcccgctgctc	cgccgttttctttgttcgtatc
BGLU Dm	tggagggcatgcactcacttc	ggtccgaaaatcggcgaagttcc
AflIII	agctgtagatctaaaaggcggccg	atagcagctggcgtggaattggc
GstD2 Dm	ccggatcggatgaggacttg	ttcgaacgtggagacagtgg
GstD5 Dm	tctattactcgccccgtgga	tctccaccagatagacggca
GstD9 Dm	agaagcttgaaaatctctcagcg	gcatccagatccacctgctt
GstE4 Dm	gtcagtgctcttccagggac	agcaccgatcctgggatact
GstE7 Dm	cgagtccggagtgatcttcg	tggtcgtgtccacctttacg
GstE9 Dm	tgtacggcgtagaggctagt	aaatggcgtgactctcccag
Afg3L2 Dm	agaaccgaaaaaccgccgta	ctgtttgggatctgctgggt
Spg7 Dm	gaccaggtggtgcagctaat	cgtgtcggtgcttaggttct

Other Supplementary Materials for this manuscript include the following:

Supplementary Movie S1: 3D reconstitution of the electron tomogram presented in Figure 5C.

Supplementary Movie S2: 3D reconstitution of the electron tomogram presented in Figure 5E.

Supplementary Movie S3: 3D reconstitution of the electron tomogram presented in Figure 5F.

Supplementary Data S1: Dataset for the proximity proteome of mitochondria and endosomes in the steady state.

Supplementary Data S2: Dataset for the proximity proteome of mitochondria and endosomes in cells expressing TWNK^{K319E}-Cherry.

Supplementary Data S3: Dataset for transcriptome analysis of the epidermis in control and Epi-ΔmtDNA larvae.

Uncropped WB and Agarose Gels

Figure 2D

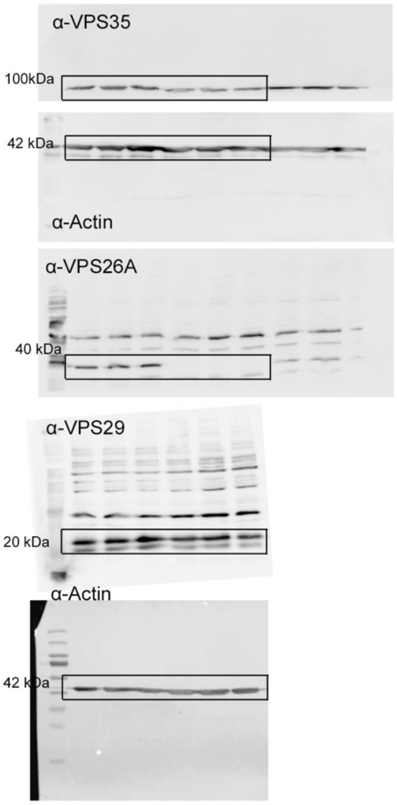

Figure 2F

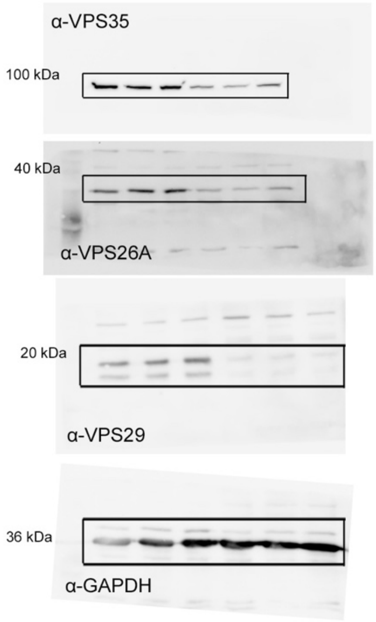

Figure 2H

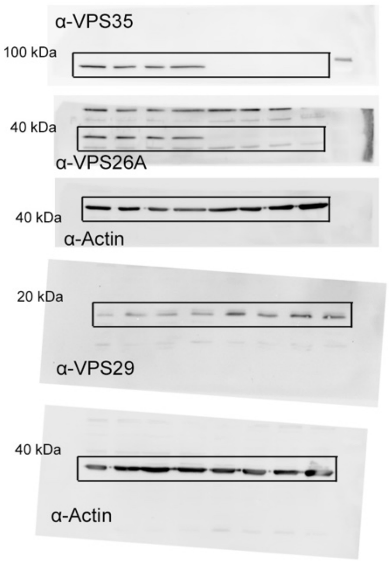

Figure 6I

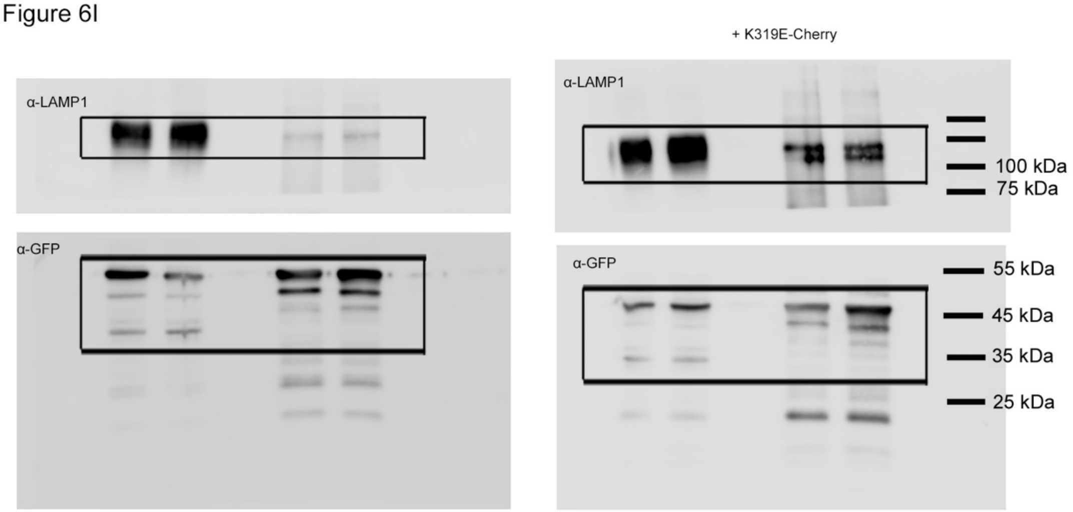

Figure 7E

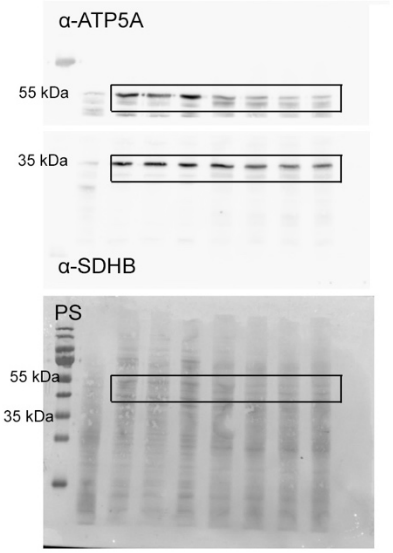

Figure S1A

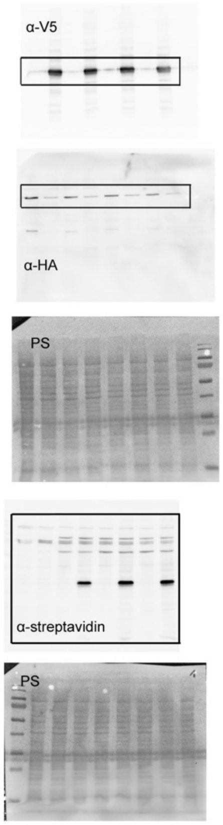

Figure S3G

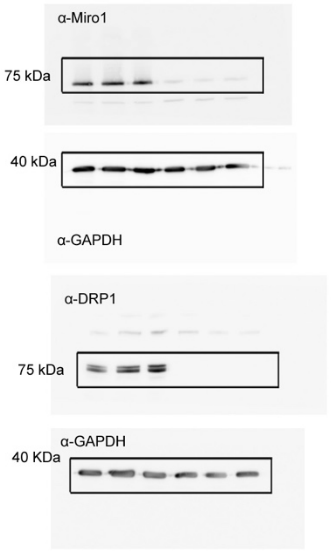

Figure S4A

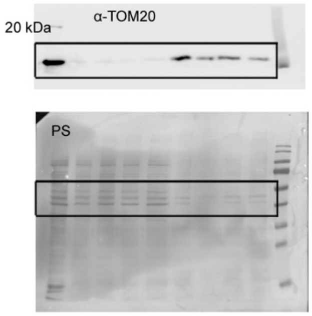

Figure S6A

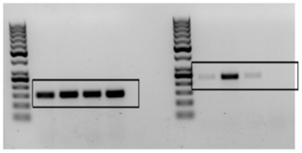

Figure 6B

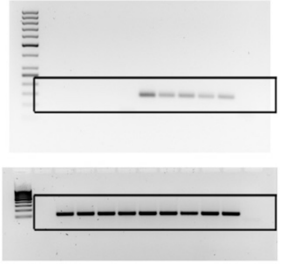

Figure S6G

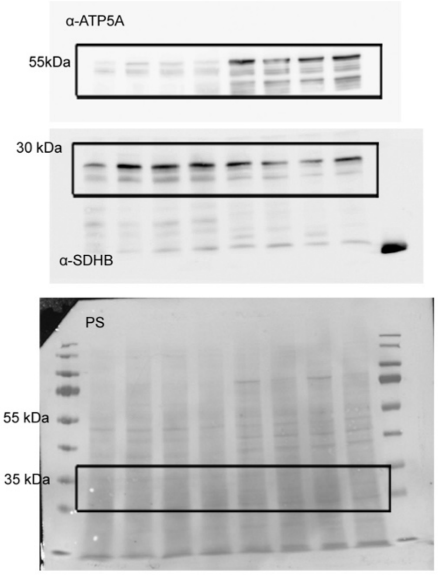

Figure S6I

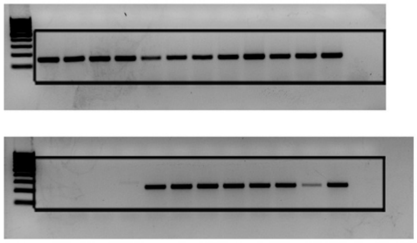

Figure S8E

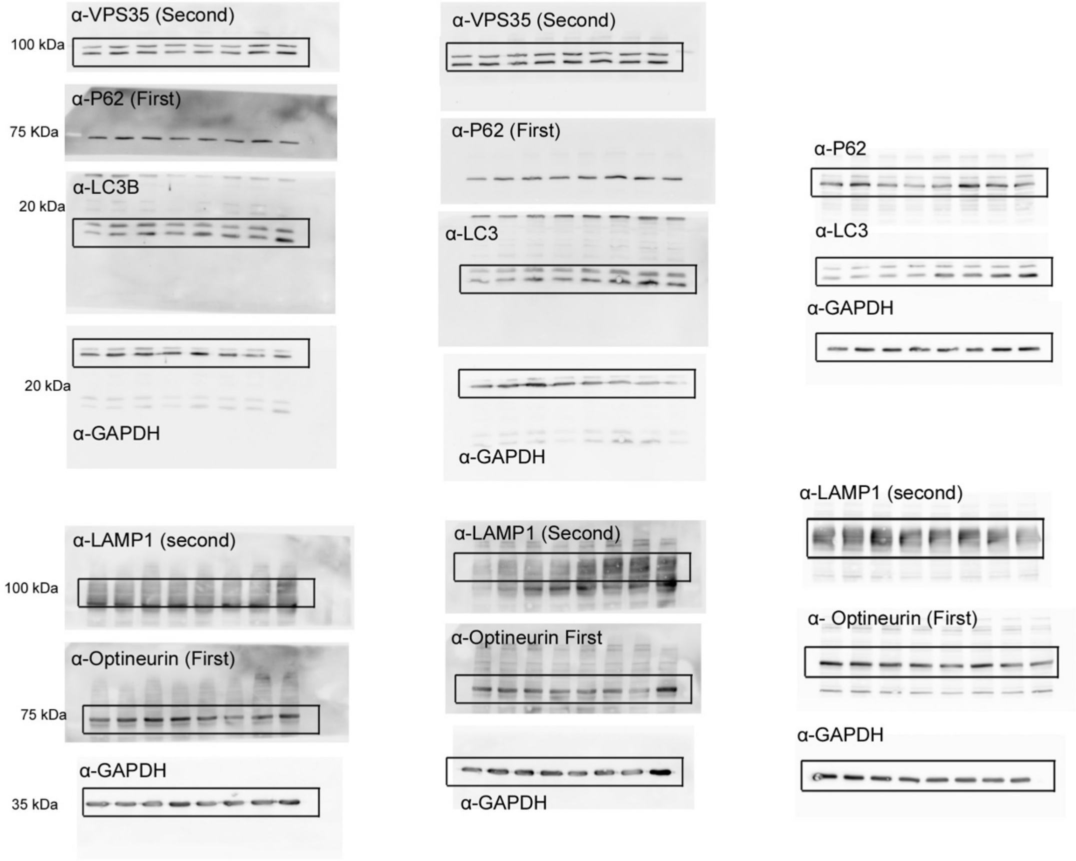

Figure S8C

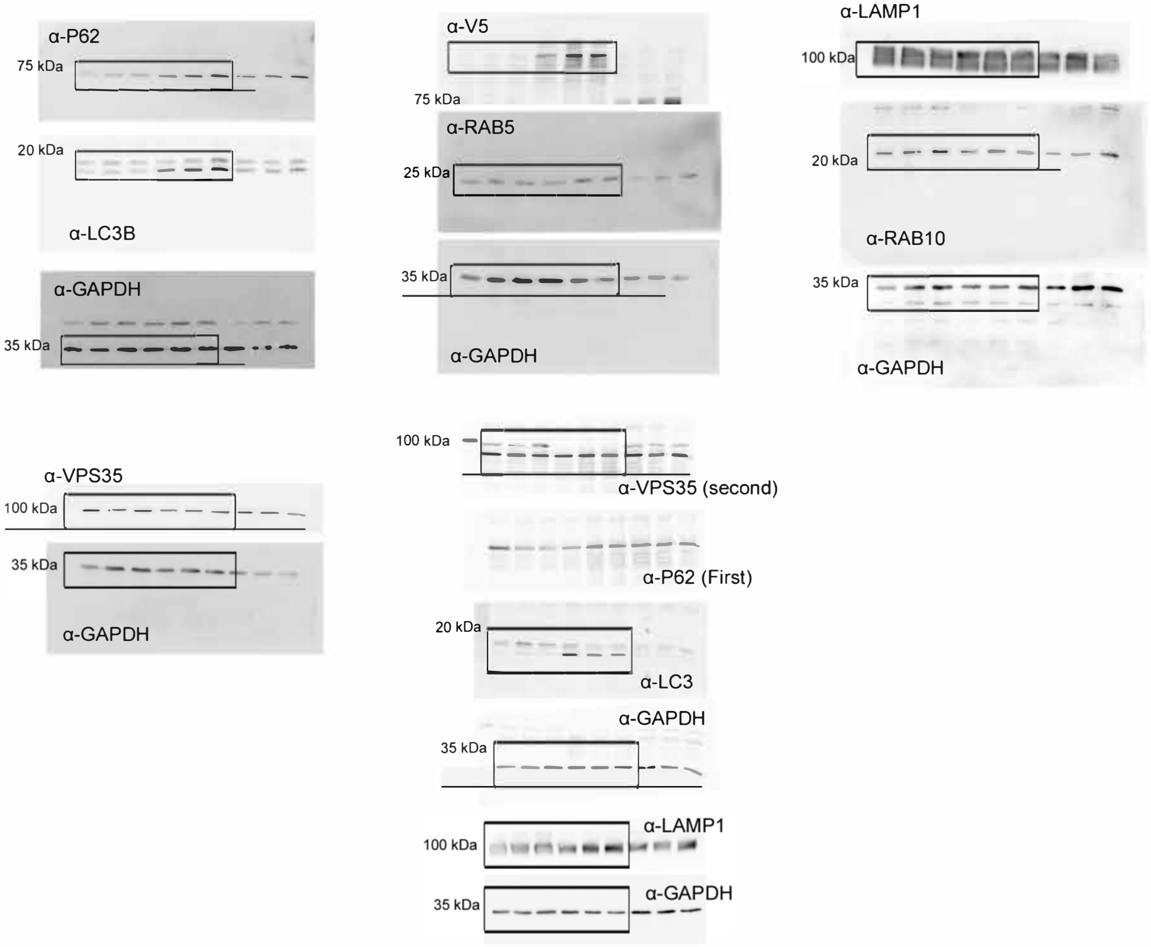
α-P62
75 kDa
20 kDa
α-LC3B
α-GAPDH
35 kDa
α-V5
75 kDa
α-RAB5
25 kDa
35 kDa
α-GAPDH
α-LAMP1
100 kDa
20 kDa
α-RAB10
35 kDa
α-GAPDH
α-VPS35
100 kDa
35 kDa
α-GAPDH
100 kDa
α-VPS35 (second)
α-P62 (First)
20 kDa
α-LC3
α-GAPDH
35 kDa
α-LAMP1
100 kDa
35 kDa
α-GAPDH
